# Methylated H3K4, a Transcription-Associated Histone Modification, Is Involved in the DNA Damage Response Pathway

**DOI:** 10.1371/journal.pgen.1001082

**Published:** 2010-08-26

**Authors:** David Faucher, Raymund J. Wellinger

**Affiliations:** Department of Microbiology and Infectious Diseases, Faculty of Medicine, Université de Sherbrooke, Sherbrooke, Québec, Canada; The University of North Carolina at Chapel Hill, United States of America

## Abstract

Eukaryotic genomes are associated with a number of proteins such as histones that constitute chromatin. Post-translational histone modifications are associated with regulatory aspects executed by chromatin and all transactions on genomic DNA are dependent on them. Thus, it will be relevant to understand how histone modifications affect genome functions. Here we show that the mono ubiquitylation of histone H2B and the tri-methylation of histone H3 on lysine 4 (H3K4me3), both known for their involvement in transcription, are also important for a proper response of budding yeast cells to DNA damaging agents and the passage through S-phase. Cells that cannot methylate H3K4 display a defect in double-strand break (DSB) repair by non-homologous end joining. Furthermore, if such cells incur DNA damage or encounter a stress during replication, they very rapidly lose viability, underscoring the functional importance of the modification. Remarkably, the Set1p methyltransferase as well as the H3K4me3 mark become detectable on a newly created DSB. This recruitment of Set1p to the DSB is dependent on the presence of the RSC complex, arguing for a contribution in the ensuing DNA damage repair process. Taken together, our results demonstrate that Set1p and its substrate H3K4me3, which has been reported to be important for the transcription of active genes, also plays an important role in genome stability of yeast cells. Given the high degree of conservation for the methyltransferase and the histone mark in a broad variety of organisms, these results could have similar implications for genome stability mechanisms in vertebrate and mammalian cells.

## Author Summary

Over the last years, it has become evident that chromatin plays a crucial role in a variety of cellular processes. Insights into the tight regulation of chromatin opening and closing via chromatin remodelers, as well as post-translational modifications of proteins making up chromatin, have revealed new facets on the mechanisms used by cells in order to replicate, transcribe, or repair their DNA. In this report, we describe the involvement of a transcription-linked histone modification, methylation of histone H3 on lysine 4, in the DNA damage repair process. We discovered that in addition to its presence at promoters of highly transcribed genes, H3K4me3 is recruited to sites of newly created double-stranded breaks. Moreover, the results show that this recruitment is dependent on a chromatin remodeler, namely the RSC complex. Cells lacking this histone modification display similar defects as those devoid of the RSC complex; i.e. a significant decrease in the repair of DNA breaks by the non-homologous end-joining repair pathway and a difficulty to survive in presence of replication stresses. All these observations highlight the importance of this conserved histone modification, given that it is involved in a variety of mechanisms affecting genome function.

## Introduction

Genomes of living organisms are continuously exposed to DNA damaging agents such as ultraviolet light, ionizing radiation, reactive oxygen intermediates or other chemical mutagens. These agents can cause mutations and ultimately genome instability. For mammalian cells, it has been estimated that endogenous DNA damage occurs at a rate of 10 000 events per day [1]. Of the observed DNA damages, double strand breaks (DSBs) potentially are the most deleterious and therefore must be efficiently detected and repaired. Eukaryotic cells have developed multiple mechanisms in order to respond adequately to such DNA damage and many of those mechanisms are highly conserved [1]–[3]. In *Saccharomyces cerevisiae,* the appearance of DSBs triggers DNA damage checkpoints which ultimately lead to arrest of the cell cycle [4]. It is thought that this controlled arrest provides sufficient time for the repair machinery to locate the break and properly repair the lesion by one of two major DSB repair pathways. During S and G2/M phases of the cell cycle, DSBs are primarily repaired by homologous recombination (HR) between sister chromatids or homologous chromosomes. In G1 and if there is no homology available elsewhere, non-homologous end joining (NHEJ) can repair DSBs by direct religation of the break. This pathway however is much more discrete in yeast.

The repair of DNA damage has to occur in the context of chromatin and its local composition, modification and configuration does affect DNA repair [5]. Therefore, DNA compaction by chromatin must be tightly and locally regulated in order to allow access of protein complexes associated with DNA transactions. There are two key mechanisms to regulate relative opening and closing of chromatin for example at a site of DNA damage: chromatin remodelling and histone modifications [6]–[9]. Chromatin remodelling is achieved by dedicated complexes that hydrolyse ATP in order to remove or mobilize nucleosomes in the area of a DSB (SNF2-type ATPases, see [10], [11], for reviews). It is thought that this creates a discrete change in the general chromatin architecture that allows more efficient repair [12]. In budding yeast, a number of chromatin remodelers such as Ino80, RSC, Swi/Snf and Swr1 have been associated with the DSB repair process [8], [13]–[18]. These complexes are rapidly recruited to a DSB and some directly interact with the DNA damage repair machinery.

Modifying histone proteins is a second possibility by which cells can change the chromatin at the site of a DSB [6], [9], [19]. Histones can be the target of a number of covalent modifications, including acetylation, phosphorylation, methylation, ubiquitination, ADP-ribosylation and sumolyation [9], [19]. An example of such a modification is the phosphorylation of histone H2A on residue S129 which can be documented very rapidly after the formation of even a single DSB [20]. A histone modification involved in a number of chromatin-regulated pathways in yeast is the ubiquitination of histone H2B on lysine 123 (H2BK123ub) [21]. This modification in fact is a necessary prerequisite for the methylation on K4, K36 and K79 of histone H3 [22]–[24]. Methylation of K79 by Dot1p has been associated with DNA damage resistance and the ability to induce the DNA damage checkpoint [25], [26]. Tri-methylation of H3K4 (H3K4me3) by Set1p on the other hand is found in the 5′-area of many highly transcribed genes and is thought to be a marker for recent transcriptional activity ([27]–[29], reviewed in [30]). A possible association of Set1p with DNA repair has been invoked ([26], [31], reviewed in [32]), but no definitive evidence has emerged. Furthermore, it has been shown that the Dot1p-mediated effect on DNA repair is distinct and separate from any possible Set1p effect [25].

Here, we show that H3K4 methylation by Set1p indeed is an important event for efficient DNA DSB repair. Consistent with previous results, Bre1p- and Lge1p-dependent ubiquitylation of H2BK123 is also required. The results show that Set1p as well as H3K4me3 accumulate at an inducible DNA DSB and the appearance of Set1p is dependent on the presence of the RSC-complex. In addition, *set1*Δ cells are deficient in the NHEJ repair pathway and appear to be impaired in traversing S-phase in the presence of replication stress. These data thus show that H3K4 methylation is associated with modulating chromatin at the site of DNA damage and significantly broadens the functional importance of this modification.

## Results

### A strong synthetic interaction between LGE1/BRE1 or SET1 and the MRX complex

**Figure 1:**
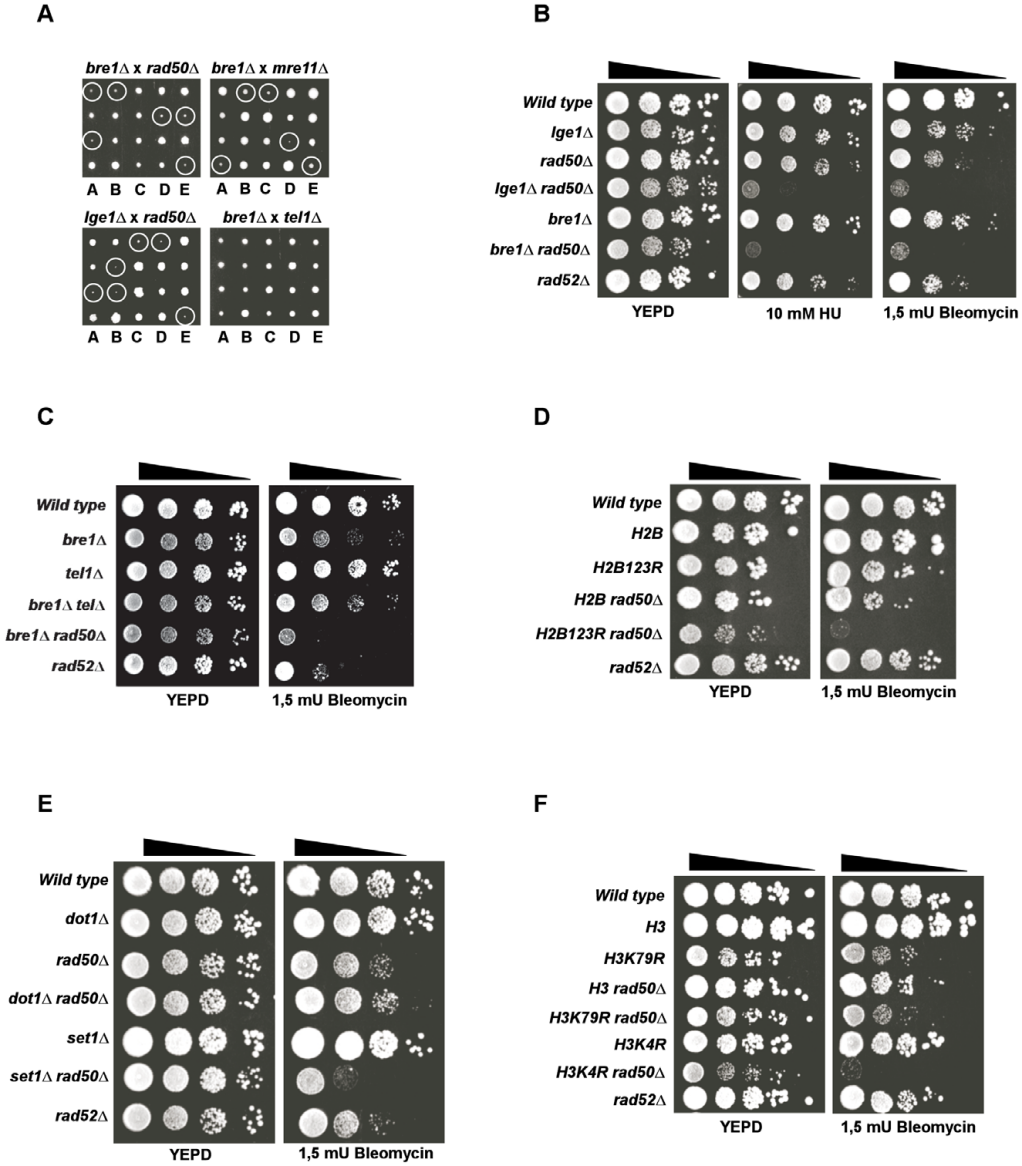
Genetic interactions between a histone modification pathway and the MRX complex. (A) Tetrad analysis of spores derived from diploids micro-dissected on YEPD plates and allowed to grow for 3 days at 23°C. Circled colonies are double mutants. Diploids DFY001, DFY002, DFY004 and DFY005 respectively, were used as starting strains. (B–F) Serial ten-fold dilution growth tests of exponentially growing cultures on plates with the indicated compounds. YEPD: control plates without drug; (B) Growth of cells with a deletion of members of the Ubiquitin-Ligase complex in combination with a deletion of *RAD50* (spores derived from diploids DFY002 and DFY007); (C) Spot dilution test of *bre1*Δ *tel1*Δ double mutant cells on plates containing the indicated concentration of Bleomycin (right) or no drug (left, spores were derived from DFY005); (D) Growth of cells with a mutant that cannot be mono-ubiquitylated (H2BK123R) (strains YZS276, YZS246, DFY008, DFY009 and MT0-73); (E) Growth of cells with deletions of *DOT1* or *SET1* genes in combination with absence of *RAD50* (spores derived from diploids DFY010 and DFY012); and (F) Growth of cells with a mutant histone H3 that cannot be methylated (H3K79R or H3K4R) and combined with an absence of *RAD50* (strains YZS267, DFY013, DFY015, DFY016, DFY017 and DFY018).

In an attempt to find potential new regulators of telomere homeostasis, a synthetic genetic array experiment was performed using *mre11*Δ and *rad50*Δ mutants as baits and looking for lethality or growth delay in double mutants [33]. The screen yielded, *lge1*Δ (YPL055C) as displaying a synthetic slow growth phenotype at 23°C when combined with the deletion of any member of the *MRE11*/*RAD50*/*XRS2* (MRX) complex (Figure 1A). Lge1p has been shown to interact genetically and biochemically with the RING finger domain protein Bre1p [23], [34]. Yeast Lge1p and Bre1p thus have been suggested to form an ubiquitin ligase complex (E3) that binds to Rad6p (E2) in order to monoubiquitylate histone H2B [23], [35]. Given these interactions, we asked if *BRE1* would display the same genetic interaction with *MRX* as *LGE1*. Indeed, double mutant *bre1*Δ *MRX*Δ cells display a very similar synthetic slow growth phenotype as *lge1*Δ *MRX* (Δ cells (Figure 1A). Interestingly, both *lge1*Δ *tel1*Δ and *bre1*Δ *tel1*Δ mutants do not display such synthetic slow growth (Figure 1A), suggesting that for this particular phenotype, *TEL1* is in a different genetic pathway than MRX. This contrasts with the fact the MRX complex and *TEL1* have been shown to be in the same genetic pathway with respect to telomere maintenance and DNA damage checkpoints [36], [37]. These experiments indicate that both Lge1p and Bre1p act in a genetic pathway parallel to MRX and which is important for normal growth of the cells.

In the course of these genetic analyses, we also discovered that *bre1*Δ *rad50*Δ cells were highly sensitive to DNA damaging agents. Both *lge1*Δ *rad50*Δ and *bre1*Δ *rad50*Δ display dramatically reduced viability when treated with the IR-mimetic drug bleomycin, UV light or the DNA replication inhibitor hydroxyurea (HU) (Figure 1B and data not shown). Note that the conditions used for inducing replication stress or DNA damage were relatively mild such that in these experiments, even *rad52*Δ or *rad50*Δ cells display only a minor growth defect as compared to a wild type strain (see Figure 1B, for example). Consistent with the observed growth properties at 23°C, a hyper-sensitivity to DNA damage was not observed in *bre1*Δ *tel1* (Δ double-mutants (Figure 1C). These data thus suggest that in the absence of MRX, DNA damage survival is dependent on the presence of *LGE1* and *BRE1*.

Previously, the *BRE1* and *LGE1* genes had been characterized as key players for modulation of gene transcription in yeast (reviewed in [21]). These genes, together with *RAD6*, are required for mono-ubiquitylation of histone H2B on the lysine residue 123 which in turn is required for the methylation of histone H3 on lysines 4 and 79 [21], [23], [35]. Set1p and Dot1p are the two methyltransferases that methylate, respectively, histone H3K4 and H3K79. Given this known cascade of histone modifications, the downstream effect of the absence of Bre1p or Lge1p could be dependent on this histone modification pathway or occur via a new and histone-independent pathway. We therefore tested whether an absence of histone modifications also sensitizes *MRX*Δ cells to DNA damage. Cells harbouring a combination of *rad50*Δ and a mutant allele of histone H2B that cannot be ubiquitinated (*H2BK123R*) are as sensitive to bleomycin as *bre1*Δ *MRX*Δ or *lge1*Δ *MRX*Δ cells (Figure 1D). Therefore, the DSB sensitivity of *bre1*Δ *MRX*Δ cells can be explained by the incapacity of the cells to ubiquitinate histone H2B on lysine 123. Further, in *MRX*Δ cells, neither the deletion of *DOT1* nor the mutation of its substrate histone H3K79 sensitizes the cells to bleomycin (Figure 1E and 1F). On the other hand, deletion of the methyltransferase *SET1* or the presence of a H3 allele that cannot be methylated on lysine 4, H3K4R, combined with an absence of the MRX complex lead to an indistinguishable sensitivity as observed in *lge1*Δ*/bre1*Δ *MRX*Δ cells (Figure 1E and 1F). *BRE2* and *SPP1* have also been reported to be required for methylation of H3K4, albeit to varying degrees for the bi-methylated and tri-methylated forms [38], [39]. However, in our hands only a deletion of *BRE2* but not that of *SPP1* lead to a complete lack of H3K4me3 (Figure S1A, S1B, and S1C). Remarkably, the sensitivity of the respective double mutants (i.e. *bre2*Δ/*rad50*Δ or *spp1*Δ/*rad50*Δ) to Bleomycin correlated with this finding: a *bre2*Δ/*rad50*Δ strain in which H3K4me3 levels are below detection as in a *set1*Δ/*rad50*Δ strain is exquisitely sensitive, whereas the *spp1*Δ/*rad50*Δ strain is not (Figure S1A, S1B, and S1C). Finally and as expected, *set1*Δ and H3K4R appear to act in the same genetic pathway (see below Figure 5A). We conclude that in the absence of the MRX complex, Set1p and the methylation of H3K4 are important for the survival of the cells in presence of DNA damage.

### Set1p-mediated H3K4 methylation is involved in DSB repair by NHEJ

**Figure 2:**
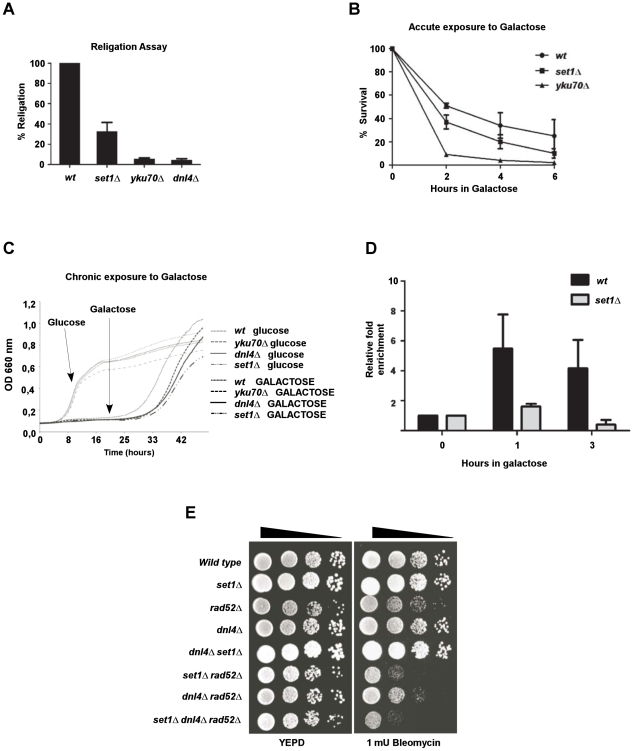
The *LGE1-BRE1-SET1* histone modification pathway is involved in DSB repair by non-homologous end joining (NHEJ). (A) *Set1*Δ cells display a deficiency in a plasmid religation assay. Linearized plasmid pRS316 was transformed into wild type cells (*wt*, YW1276), *set1*Δ (DFY021), *yku70*Δ (YW1283) or *dnl4*Δ cells (DFY022). Overall percentage of religation was calculated using transformation efficiency normalization. (B) Survival of *wt* (JKM179), *set1*Δ (DFY023) or *yku70*Δ cells (JKM181) following induction of a DSB. Cells were held in galactose media for the indicated time and then plated on glucose medium, shutting off HO endonuclease expression. In these strains, the induced DSB at the mating type locus can only be repaired by NHEJ. (C) Growth curves of *wt* (YW1276), *set1*Δ (DFY021), *yku70*Δ (YW1283), and *dnl4*Δ cells (DFY022) exposed to a constant expression of HO endonuclease. (D) Recruitment of Yku80-myc protein to an induced DSB site in *wt* (DFY048) and *set1*Δ (DFY049) cells. DSB induction was performed for 1 and 3 hours, ChIP performed with an anti-myc antibody and immunoprecipitated DNA quantified by Q-PCR. Fold enrichment is normalized to non-induced cells (glucose) set as 1. (E) Sensitivity to DNA damage of cells harbouring deletions of genes affecting, respectively, NHEJ (*DNL4*) or homologous recombination (*RAD52*) when combined with a deletion of *SET1*. Strains used were spores of DFY020.

Because cells lacking both *SET1* and the MRX complex are very sensitive to DSB, we decided to verify which of the main DSB repair pathways, homologous recombination (HR) and non-homologous end joining (NHEJ) were affected in cells lacking Set1p. In order to test whether *SET1* indeed is involved in NHEJ, *set1*Δ-cells were analyzed for NHEJ efficiency. A plasmid religation assay was performed using a transformation assay with linearized vector DNA. The circularization efficiency in strains deleted for *YKU70* and *DNL4* was about 5% of that obtained in *wt* cells, confirming their importance in this assay (Figure 2A). Notably, the absence of *SET1* also caused an important decrease in the capacity of cells to complete NHEJ and to religate the plasmid: overall religation efficiency in these cells was reduced to about 30% of that seen in *wt* (Figure 2A).

In addition, we used strain JKM179 harbouring the HO endonuclease controlled by a galactose-inducible promoter. In this strain, the natural donor sequences usually used for homologous recombination after the HO recognition site is cut are deleted from the genome. Therefore, upon induction of expression of the HO endonuclease, these cells can only repair the DSB at the mating type locus by NHEJ [40]. Deletion of *SET1* in this strain background and exposing the strains to galactose resulted in a significant decrease of the viability of the cells as compared to *wt*, albeit not to the level of sensitivity of *yku70*Δ cells that display the expected dramatic decrease in viability due to an almost complete absence of NHEJ (Figure 2B).

To confirm the involvement of *SET1* in the NHEJ repair pathway, we also used a strain that contains a cassette flanked by two *HO* cleavage sites [41], [42]. Following *HO* endonuclease induction by addition of galactose to growth media, both *HO* cleavage sites are cut and given the absence of homology in the rest of the genome for sequences on either side of the cassette, the gap had to be repaired by NHEJ (see Figure S2A). When grown in liquid culture containing galactose as carbon source and thus continuously induced HO cleavage, strains with a deletion of *SET1* display a similar extended lag-time before growth recovery as strains deleted for *YKU70* or *DNL4* (Figure 2C). In these conditions, growth recovery could be dependent on two different mechanisms: error-prone NHEJ or MMEJ (Microhomology-mediated end joining). In the case of wild type and *set1*Δ cells, growth is restored via the error-prone NHEJ during which the HO-recognition site must be lost such the HO enzyme does not continuously induce the DSB. However cells completely unable to carry out NHEJ (cells harbouring *yku70*Δ and *dnl4*Δ alleles), bypass the continuous HO cleavage via MMEJ using small stretches of homology in order to perform DSB repair [43]. The results obtained with the liquid culture assays were paralleled by results obtained with the same strain, but using a colony color assay for error-free and error-prone assays ([41], [42], Figure S2B). Given the dependence of NHEJ on the presence of the Yku-proteins at the DNA break, it was possible that in Set1p-lacking cells, the binding of Yku to the break was impaired. We used chromatin immunoprecipitation (ChIP) experiments with a myc-tagged Yku80p to assess Yku binding at the induced break at the *MAT*- locus and the results show significantly reduced association of Yku with the breaks (Figure 2D). In parallel experiments, the same experimental setup with gal-induced expression of the HO endonuclease was used to assess HO-cutting efficiencies of the HO-sites flanking the *ADE2-*cassette and on a plasmid borne *MAT*-locus (see Figure S2A, S2C, and S2D). The results confirm that these induced cleavages occur at indistinguishable rates when wild-type and *set1*Δ cells are compared (Figure S2C and S2D), a result that is also consistent with the sensitivity to HO-induced DSB of the *set1*Δ cells (Figure 2C). These experiments establish that in *set1*Δ cells, the efficiency of NHEJ, in particular of the error-prone branch of this repair pathway, is impaired when compared to wild type cells. Consistent with this conclusion, Yku-binding at an induced DSB is reduced.

Abolition of the two DSB repair pathways (NHEJ and HR) at the same time leads to synergetic sensitivity of the cells to DNA damaging agents. As an example, this can be observed for cells harbouring deletions of both *DNL4* and *RAD52* and in which NHEJ and HR are lacking ([44], Figure 2E). Thus, we combined deletions of *DNL4* and/or *RAD52* with *set1*Δ and tested the sensitivity of the resulting cells to bleomycin. At the bleomycin concentration used in this experiment, neither *set1*Δ nor *dnl4*Δ single mutants display a significant sensitivity and grow as well as wild type cells. As mentioned above, r*ad52*Δ cells were only mildly affected (Figure 2E). The *set1*Δ *dnl4*Δ double mutant cells also were not sensitive to this bleomycin concentration, which is consistent with the possibility that these two genes act in the same genetic pathway. Yet, double mutant *set1*Δ *rad52*Δ and particularly the triple mutant *set1*Δ *dnl4*Δ *rad52*Δ displayed a sensitivity to DSB that is even higher than that of *dnl4*Δ *rad52* Δ cells (Figure 2E). However, as assessed by a Q-PCR method, we could not discern in *set1*Δ cells a major defect in homologous recombination mediated mating-type switching (Figure S3), which is consistent with the observation that in the presence of the HR machinery, *set1*Δ cells are not very sensitive to radiomimetic drugs (Figure 1E and Figure 2E). These results confirm that *SET1* is involved in the NHEJ repair pathway and suggests that it may even have additional roles in maintaining genome stability, particularly in the presence of DNA damage.

### Recruitment of the SET1 methyltransferase and detection of H3K4me3 at newly created DSBs

**Figure 3:**
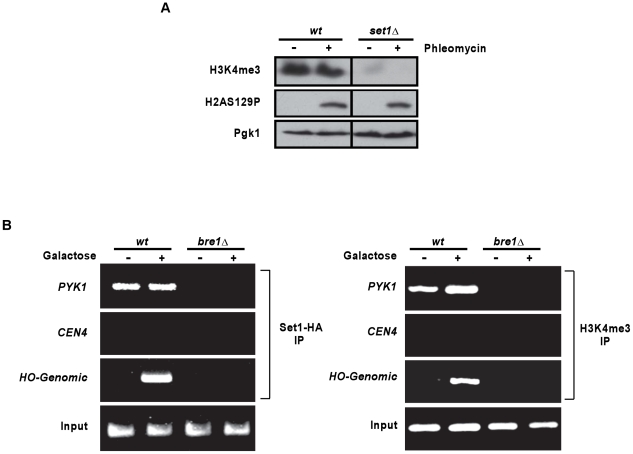
Site-specific recruitment of the Set1p methyltransferase and detection of H3K4me3 at a DSB. (A) Western blot of proteins derived from *wild type* and *set1*Δ cells that were treated (+) or not (−) with phleomycin. Top: Tri-methylated H3K4 was detected with an antibody against H3K4me3; middle: detection of phosphorylated H2AS129 as a positive control for phleomycin treatment; and bottom: anti-Pgk1p antibody as a loading control. Strains used were *wt* DFY024 and *set1*ΔDFY026). (B) Left: Recruitment of HA-tagged Set1p methyltransferase to the mating type locus was tested by ChIP analysis using an anti-HA antibody. HO endonuclease was expressed for 90 min in G1-arrested *wt* (DFY027) or *bre1*Δ cells (DFY028). DNA was extracted from immunoprecipitates and PCR amplified with specific primers adjacent to the HO cleavage site, primers in the promoter region of the highly expressed gene *PYK1,* or primers specific for the transcriptionally repressed *CEN4* locus. Right: Same experiment as in left panel, except that instead of the anti-HA antibody, an antibody specific for H3K4me3 was used for the immunoprecipitation.

It is well documented that H3K4 trimethylation is present on H3 in nucleosomes at a majority of promoters and 5′ parts of active genes [27], [28], [30], [32], [45], [46]. Based on the observations above highlighting the importance of H3K4me3 in DNA damage repair processes, we asked whether or not this histone modification could be induced at the site of DNA damage. First, we tested whether there was a global increase in H3K4me3 in chromatin following DNA damage. Exposure to phleomycin of wild type cells did not result in an increase in the global amount of H3K4me3 (Figure 3A and Figure S4). Moreover, the levels of phosphorylated H2AS129 did not significantly vary between *set1*Δ cells and wild type cells, indicating that this part of the chromatin modification at a DNA damage site seems to be by en large properly activated in this mutant (Figure 3A). The failure to be able to detect an increase in H3K4me3 after DNA damage induction by phleomycin could be explained by an already relatively high level of H3K4me3 that is associated with active transcription. A slight further increase of that global level in this experiment may simply be undetectable by western blotting, even if high doses of phleomycin were used (Figure S4). We therefore turned to an assay that verifies the potential recruitment of the Set1p methyltransferase to a specific DSB by (ChIP) analysis. Fortunately, Set1p and H3K4me3 are virtually absent from the endogenous HO cut site located at the mating type locus ([23]; Figure 3B). In our setting, the HO endonuclease was induced in wild type or *bre1*Δ cells, both also harbouring a functional Set1p-HA allele. Chromatin was immunoprecipitated with an anti-HA antibody [23] or a H3K4me3-specific antibody. As expected, irrespective of whether HO is induced or not, Set1p and H3K4me3 can be detected on the promoter of *PYK1*, a gene known to be highly expressed throughout the cell cycle (Figure 3B, [23]). On the other hand, neither was detectable at the centromere of chromosome 4, an area that is transcriptionally repressed in a constitutive fashion. Finally, when the cells were grown in glucose and in the absence of the HO endonuclease, Set1p and H3K4me3 were not detected at the MAT locus, while after induction of HO by galactose; both became detectable at this same locus (Figure 3B). Formally, it was possible that addition of galactose to growth media leads to a transcription mediated recruitment of Set1p. However, as assessed by Northern blotting and RT-QPCR, the level of Mat**a**1-RNA transcribed at the *MAT* locus in these cells did not vary significantly between glucose and galactose-grown cells (Figure S5A and S5B). Furthermore, we also assessed occurrence of H3K4me3 at *MAT* in a strain containing a *MAT* locus that is not susceptible to HO-cutting (*MATa-inc*) by ChIP and no increase of the H3 modification could be detected (Figure S5C). These results therefore show that Set1p associates with a newly created DSB *in vivo* and induces *de novo* trimethylation of histone H3 at the site.

### Intra-S retention of set1Δ mutants following exposure to DNA damaging agents

**Figure 4:**
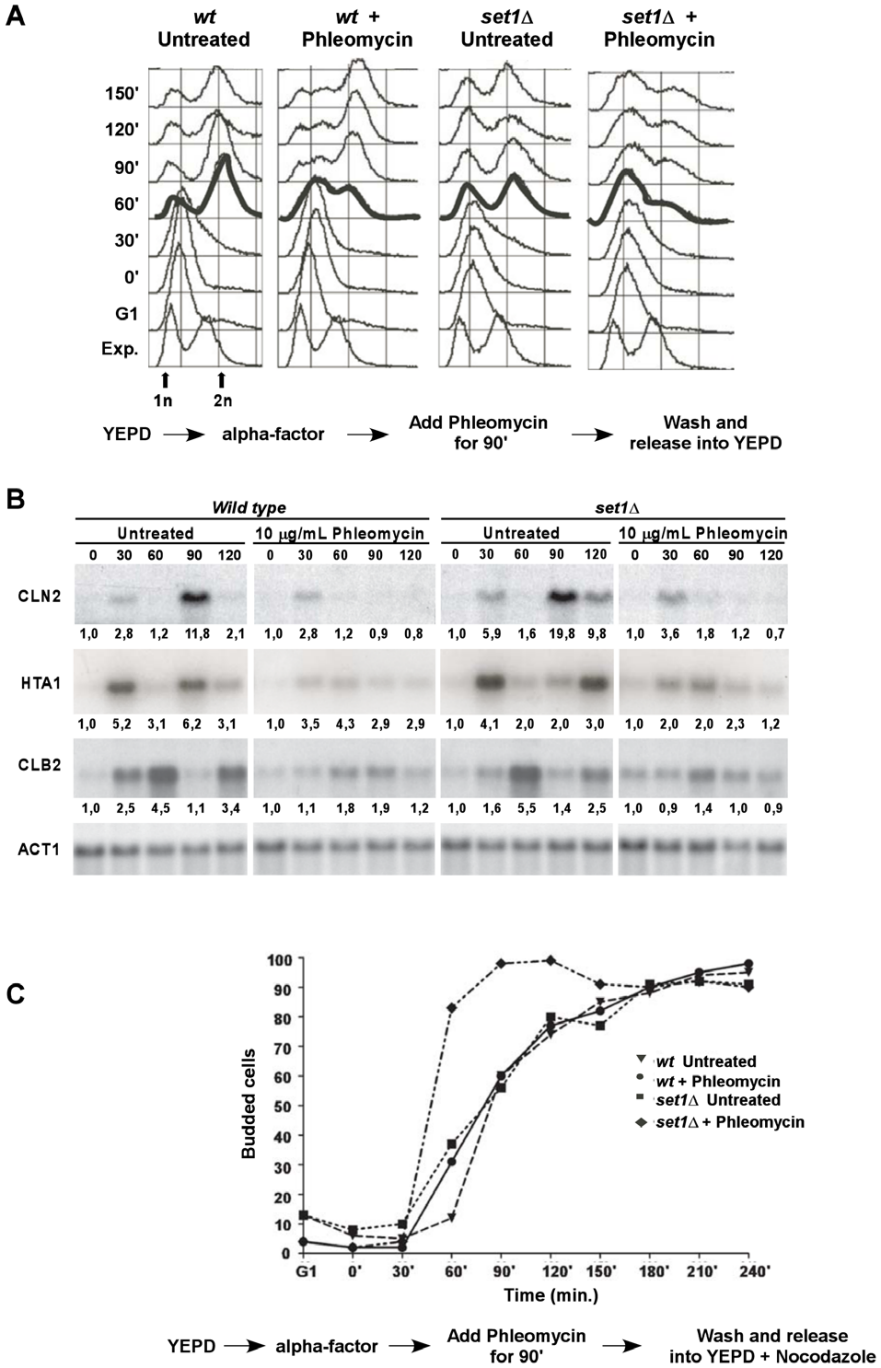
*Set1*Δ cells exposed to DNA damage in G1 are retained in S-phase. (A) *wt* (DFY024) and *set1*Δ cells (DFY026) were arrested in G1 phase of the cell cycle and exposed to phleomycin (see experimental scheme at bottom). Cells were then released and cell cycle progression was monitored by flow cytometry of samples at regular intervals. Time 0 min represents the release of cells into rich medium immediately following the phleomycin treatment. (B) In a separate release experiment, northern blot analysis was performed in order to confirm the expression of cell-cycle specific mRNAs: Cln2 for G1, Hta1 for S-phase and Clb2 for G2/M specific RNA expression. A probe specific for the Act1-mRNA was used as a non-varying control (bottom). The number below each lane indicates the change of the signal intensity for the particular RNA; the level at time 0 was set as 1. (C) Budding indexes of *wt* (DFY024) and *set1*Δ cells (DFY026) subjected to a cell synchrony and release experiment as outlined below the curves. Note that the final media did contain Nocodazole to prevent cells to traverse multiple cycles and that explains why the indexes stay high at the end of the experiment.

In order to verify whether cell cycle transitions that are controlled by checkpoint activation are normal in mutants where H3K4 trimethylation was abrogated, we analyzed cell cycle progression of cells after exposure to DSB inducing agents (Figure 4A). Cells were arrested in G1, treated with 10 µg/ml of phleomycin for 90 min, then washed two times with sterile water in order to remove all traces of the DNA damaging agent as well as the α-mating factor and finally released into fresh media lacking drugs. Compared to untreated wild type cells, phleomycin exposed cells were delayed for 30 min before entry into S-phase, as expected (Figure 4A). However, once the G1 checkpoint delay overcome, the majority of cells resumed their cell cycle and went on to the G2/M phase. Untreated *set1*Δ-cells did also display a slight delay in S-phase entry, but the cells did resume cycling (Figure 4B). Exposure of the G1-synchronized mutant *set1*Δ to the same concentration of DNA damaging agent as above resulted in a retention of the majority of the cells in G1 or early S phase of the cell cycle (Figure 4A). These cells appeared not to resume cycling as they failed to reach the G2/M phases of the cycle (Figure 4B).

From these FACS analyses, it was not possible conclusively to deduce whether treated *set1*Δ cells remained arrested in G1 or actually did enter but could not complete S-phase. Therefore, in order to determine more precisely the exact phase of the cell cycle where these cells were arrested, we decided to analyze mRNA expression profiles of the G1/S cyclin Cln2p, the S-phase specific transcript for histone H2A (HTA1) and the G2/M cyclin Clb2p. Aliquots of cells were taken at 30 min intervals after release from the G1-arrest and mRNA was extracted. In untreated samples, both for wild type and *set1*Δ cells the expected oscillations of the expression of Cln2 or Clb2 RNA through the cell cycle can be observed (Figure 4B, top and third rows). Peak expression levels for these two cyclins are temporally separated and occur at the expected time after G1 release indicating properly cycling cultures. Following exposure to phleomycin, both wild type and mutant cells display an induction of the Cln2 RNA at 30 min whereas Clb2 RNA increases after about 60 min, but there is no detectable mRNA peak at later time points, (Figure 4B). These data are consistent with the FACS profiles as they confirm that such cells do not complete further cell cycles. The expression profiles of the S-phase specific histone H2B mRNA strongly suggest that bleomycin treated *set1*Δ cells in fact do enter S-phase and are not blocked at the G1/S boundary: Hta1 mRNA is induced at the 30 min time point and remains at an elevated level for about 60 min (Figure 4B). This induction of the Hta1 mRNA strongly suggests actual entry into S and the assessment of budding indexes during a similar experimental protocol supports this notion (Figure 4C). Collectively, these data show that cells lacking Set1p and exposed to DNA damaging agents can enter S-phase but encounter difficulty traversing it.

### Evidence for sensitivity of set1Δ cells to replication stresses

**Figure 5:**
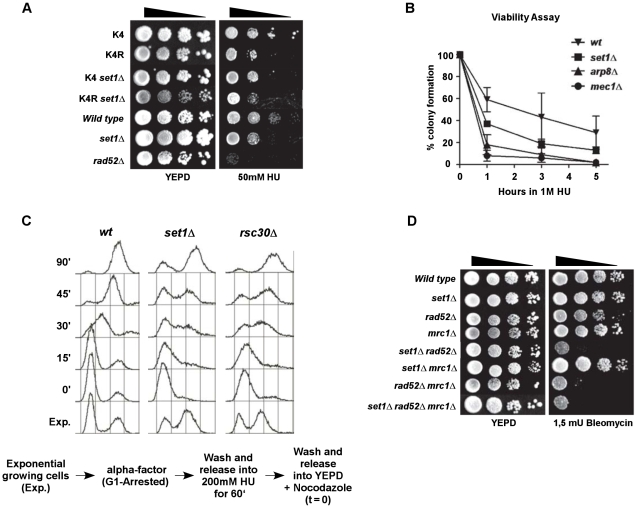
Replication defects in *set1*Δ cells. (A) Serial ten-fold dilution growth test of strains with the relevant genetic setup on plates with 50mM HU. Strains used were YZS267, DFY017, DFY038, DFY039, DFY024, DFY026 and MT0-73. (B) Viability curves of the indicated strains exposed to HU for the indicated time. Strains used were *wt* (DFY024), *set1*Δ (DFY026), *arp8*Δ (DFY030), *mec1*Δ (DFY044). (C) *wt* (DFY024), *set1*Δ (DFY026)or *rsc30*Δ (DFY029) cells were arrested in G1 phase of the cell cycle and released into a synchronous S-phase in the presence of 200 mM HU for 60 min. The HU block was removed by washout and cell cycle progression was analyzed by FACS. Time 0 min represents the release of cells into rich medium plus Nocodazole following the HU treatment. (D) Viability of strains with indicated genotype and derived from strain DFY032 were grown in liquid media and ten-fold serial dilutions were plated onto YEPD control plates or onto a plate containing bleomycin, as indicated.

The above experiments show that after genotoxic treatments, cells without the ability to introduce the H3K4me3 chromatin mark experience problems in S-phase. One hypothesis that could explain the results is that replication of damaged DNA in *set1*Δ cells is compromised. Indeed, *set1*Δ cells and cells expressing H3K4R are sensitive to hydroxyurea, a compound affecting DNA replication, confirming that tri-methylation of H3K4 is important for the passage through S in the presence of genotoxic stress (Figure 5A and 5B). The *set1*Δ, H3K4R double mutant displays a similar sensitivity as each single mutant, again consistent with the suggestion that they act in the same genetic pathway in terms of HU-sensitivity (Figure 5A). Next, we analyzed S-phase progression in wild type and *set1*Δ cells after a transient exposure to HU. Cells were arrested in G1, then released into S in the presence of 200 mM HU and finally HU was washed out and cells released into rich media containing Nocodazole (Figure 5C). In wild type cells, this situation causes some delay in S-phase resumption (data not shown), but after 30 min, most cells resumed and they completed S-phase after 45 min (Figure 5C, left). For *set1*Δ cells, the progression profile is delayed by about 15 min and many cells have not reached G2 even 90 min after the release (Figure 5C, middle). Another mutant known to enter but incapable to traverse S-phase, *arp8*Δ, a member of the INO80-complex [8], [17], [18], exposed to similar experimental conditions showed a comparable phenotype as *set1*Δ cells (Figure 5B and data not shown). Furthermore, *set1*Δ *rad52*Δ and *mrc1*Δ *rad52*Δ cells displayed a very similar sensitivity to exposure to a low dose of bleomycin, while either deletion alone or a combination of *set1*Δ *mrc1*Δ did not display any significant sensitivity (Figure 5D). Mrc1p is involved in the activation of the Rad53 kinase during the S-Phase checkpoint [47], [48] and also has an independent function in the stabilization of stalled replication forks [49]–[51]. Moreover, while both *set1*Δ and *yku70*Δ cells are compromised in their ability to repair DSBs by NHEJ, only *set1*Δ-cells, but not *yku70*Δ cells are sensitive to HU (Figure S6). These experiments strongly suggest that Set1p is important for the passage of cells through S-Phase in presence of DNA damage and replication stress and this effect is at least partially independent of the effect of a *set1*Δ on NHEJ.

### SET1 acts in conjunction with the RSC remodelling complex

**Figure 6:**
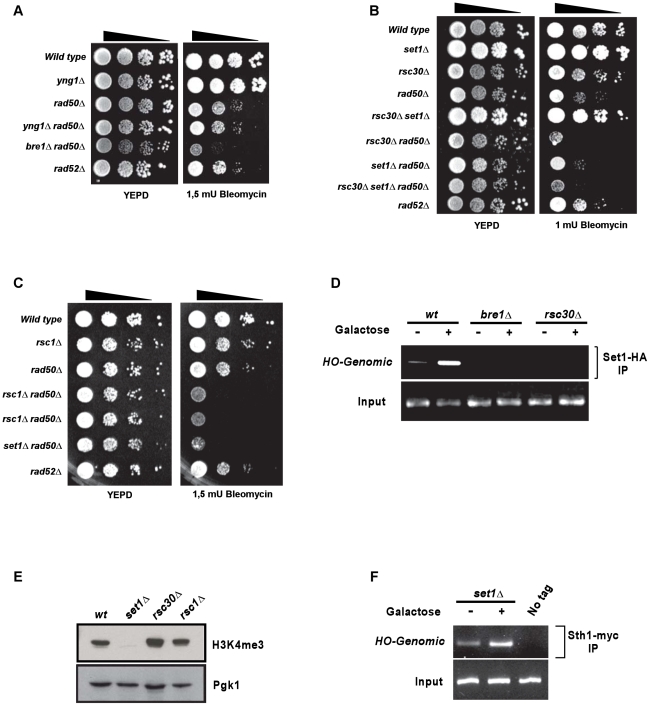
Functional interactions between the RSC complex and *SET1*. (A) Example of a spot dilution - colony viability assay performed to unc subunits *RSC30* in (B) and *RSC1* in (C), in combination with a deletion of *RAD50* and/or *SET1* (spores derived from diploid DFY034 and DFY050 respectively). Serial ten-fold dilution growth tests of exponentially growing cultures on plates containing DNA damaging agent bleomycin. YEPD: control plates without treatment. (D) ChIP experiments assessing the presence of Set1p in *wt* cells (DFY027) or in cells lacking either *BRE1* (DFY028) or *RSC30* (DFY037). (E) Western blot analysis detecting the presence of H3K4me3 in strains harbouring deletions of RSC complex subunit genes. A *set1*Δ–strain serves as negative control and re-hybridization with and anti-Pgk1p antibody was used as a loading control. Strains used were spores of diploid DFY034 and DFY050. (F) ChIP of an essential RSC-component, Sth1p, in cells lacking *SET1*. Strain used was DFY046. ChIP experiments essentially were conducted as described in Figure 3 over genes displaying a similar DNA damage sensitivity enhancement as *set1*Δ in absence of homologous recombination. Note that *yng1*Δ scored here is negative, the *bre1Δ rad50*Δ serves as positive control; strains used are spores of DFY033. (B,C) Growth of cells with a deletion of RSC complex.

The above results show that binding of Set1p and methylation at H3K4 are important events at sites of DNA damage in S-phase. Thus, we speculated that the methylated histone H3 may be important for the subsequently necessary chromatin remodelling and DNA repair activities. Methylated histones can bind or stabilize the association of various proteins that contain bromodomains, tudor domains alongside chromodomains or plant homeodomain (PHD)-finger motifs [30], [52]. Selected yeast candidate genes containing such domains and having the potential to bind H3K4me3 were tested for a genetic interaction with *RAD50,* similar to the one of the genes in H2BK123ub/H3K4me3 pathway in the presence of DNA damage. Amongst the genes tested in this fashion were *CTI6* (SAGA-complex), *CHD1* (SAGA and SLIK-complexes), *SAS3* and *YNG1* (Nua3-complex), *EAF3* (Nua4-complex), *ISW1*, *RDH54* and *RAD54*; none of which showed an interaction comparable to the one of *SET1* (Figure 6A for *YNG1* as an example). The exceptions in this regard were non-essential members of the yeast RSC remodelling complex, *RSC1* and *RSC30*. When deletions of either of those genes were combined with *rad50*Δ, the cells displayed a DNA damage sensitivity that was very similar to that of *set1*Δ *rad50*Δ cells (Figure 6B and 6C) and *rsc2*Δ *rad50*Δ double mutants also showed increased sensitivity, albeit not as dramatic (data not shown). In addition the *rsc30*Δ *set1*Δ *rad50*Δ triple mutant cells were as sensitive as each double mutant, which is consistent with the idea that *SET1* and *RSC30* act in the same genetic pathway (Figure 6B).

In order to further examine whether cells lacking *RSC30* or *SET1* behave in a similar fashion, cell cycle synchrony experiments were performed (Figure 5C). *Rsc30*Δ cells were synchronized in G1, released into a synchronous S-phase in the presence hydroxyurea for 90 min and then released by washing out the drug. As observed for *set1*Δ cells, *rsc30*Δ cells experienced an S-phase delay (compare *wt* and *rsc30*Δ cells at 45 min after release, Figure 5C). The above experiments thus establish a number of intriguing correlations between the RSC complex and Set1p: they are both associated with newly created DSBs, their absence causes strongly compromised NHEJ and also an increased sensitivity to genotoxic agents if HR is also compromised (Figure 2, Figure 3, Figure 6B, and [13]). We therefore investigated whether the recruitment of Set1p to DSBs was dependent on RSC or vice versa. As already shown above, Set1p localization to an induced DSB at MAT is readily detectable in *wt*, but abrogated in *bre1*Δ cells (Figure 3, Figure 6D). In addition, similar to the situation in *bre1*Δ cells, Set1p recruitment was undetectable in *rsc30*Δ cells (Figure 6D). This absence of Set1p recruitment to a break was not due to a general absence of the H3K4me3 modification in *rsc30*Δ cells: as assessed by western blot, the overall H3K4me3 levels remained similar to wild-type in these cells (Figure 6E), suggesting that the H3K4me3 modification associated with highly transcribed areas occurs normally in *rsc30*Δ cells. On the other hand, myc-tagged Sth1p, an essential subunit of the RSC-complex can be detected on DSBs in *wt* and *set1*Δ cells (Figure 6F and data not shown).

**Figure 7:**
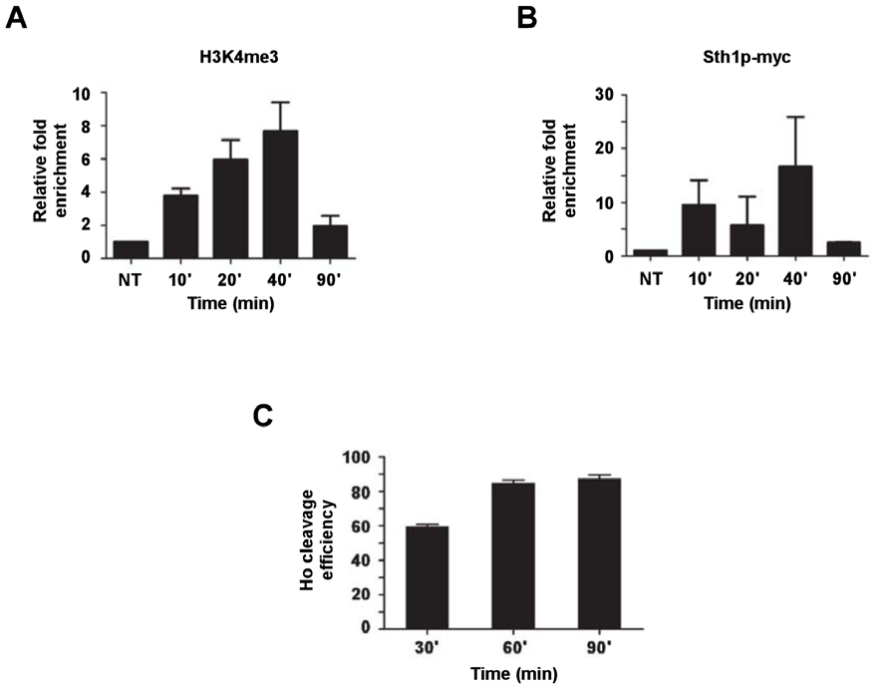
Kinetics of association of H3K4me3 and Sth1p of the RSC complex with an induced DSB. (A,B) ChIP analysis showing the recruitment of H3K4me3 and Sth1p respectively to an induced DSB. Wild-type cells (DFY046) were exposed to galactose for the indicated period in order to induce HO endonuclease. DNA was extracted from immunoprecipitates and analyzed by quantitative PCR using specific primers adjacent to the HO cleavage site. (C) Analysis of the HO cleavage efficiency of cells used for ChIP analysis in (A,B) using Q-PCR. DNA was extracted from cells and Q-PCR with primers overlapping the DSB was performed. Time 0 (glucose) was set to 0% cleavage efficiency.

In order to verify whether a temporal order of binding of the RSC-complex versus the occurrence of H3K4me3 at a DSB can be established, Sth1p-myc and H3K4me3 were immunoprecipitated at different time intervals after a DSB induction. The results show a time dependent increase for both, peaking at about 40 min after break induction (Figure 7A and 7B), but they don't allow a differentiation in terms of order of appearance. Note that after only 30 min, cleavage efficiency in this strain reached about 60% (Figure 7C).

These results are consistent with the genetic data above (Figure 6B and 6C) and strongly suggest that Set1p recruitment to a DSB is dependent on the proper function of the RSC-complex.

## Discussion

Appropriate regulation of chromatin modifications and chromatin remodelling is essential for efficient transcription, replication and repair of DNA (Reviewed in [21], [30]). The results of this study demonstrate that in budding yeast, the methylation of lysine 4 on histone H3 (H3K4me3) is involved in maintaining genome stability. Strains lacking the respective methyltransferase Set1p display three specific phenotypes that support this statement: an impaired ability to recruit the Yku-proteins to a DSB leads to a strongly reduced efficiency of NHEJ (Figure 2), *de novo* Set1p as well as H3K4me3 appearance at a site of an induced DSB (Figure 3), and a difficulty to pass through S-phase in presence of replication stresses (Figure 5). These effects, when combined with an absence of HR lead to a hypersensitivity to DNA-damaging agents (Figure 1, Figure 2, Figure 5, Figure 6). Although these observations are paralleled in strains that harbour mutations not allowing ubiquitylation of H2B (Figure 1 and Figure 5), this fact can be explained by the dependence of H3K4 methylation on previous H2B modification [53], [54]. However, while H2BK123ub is also required for H3K79 methylation by Dot1p, our data demonstrate that loss of NHEJ-efficiency and the synthetic interactions with MRX are specific to a lack of H3K4me3 and not H3K79me3 (Figure 1). Therefore, the roles played by Set1p in genome stability are quite different from those of the Dot1p methyltransferase [25], [26].

This is one of the first reports directly linking H3K4me3, a chromatin modification extensively described for its involvement in marking transcriptionally active loci, to DNA damage repair processes. Given that our results document the *de novo* occurrence of Set1p and the H3K4me3 mark on an induced DSB (Figure 3, Figure 6, Figure 7), we do not think that the effects observed in *set1*Δ-cells are indirect consequences of a change in transcriptional activity. Furthermore, analyses of microarray data of RNA derived from cells harbouring H3K4R, a mutated version of H3 that cannot be methylated, or from cells lacking either of Bre1p, Lge1p, Rad6p and Set1p do not indicate important transcription variation of genes implicated in the DNA damage checkpoints or repair [27], [55], M.-A. Osley, personal communication). For example, we could find only 5 genes for which the change was more than 3 fold (increase or decrease) in *lge1*Δ-cells (12 for *bre1*Δ cells) and none of those have a known association with DNA repair (see http://www.utoronto.ca/greenblattlab/BUR.xls). In fact, one study reported a potential increase of repair genes in the absence of Set1p, which would be the opposite effect and could not explain our results [56]. In yeast, there already is evidence for transcription-mark independent functions of Set1p. For example, a number of described subtle cellular phenotypes associated with a *SET1* deletion could be independent of transcriptional changes [57]. More recently, Set1p has been shown to methylate Ipl1p and be involved in chromosome segregation, an effect that is independent of its transcriptional regulation [58]. Finally, recent data does implicate H3K4me3 at sites of active meiotic DSB formation independently of the transcriptional state of the local genomic area [59] and there is one report that links Set1p absence with a deficiency of meiotic S-phase passage, a phenotype similar to the one reported here [60]. Intriguingly, the results on an involvement of Set1p in the regulation of meiosis in yeast have parallels in mouse, where a meiosis-specific H3K4 methyltransferase called Meisetz/*PRDM9* is required for passage through meiosis. Remarkably, meiotic cells in mice lacking Meisetz also display a strong deficiency in DSB repair [61].

Our present hypothesis stipulates that the H3K4me3 chromatin mark is crucial for adequate repair of DSBs, whether they occur accidentally during vegetative growth or are induced in meiosis. For example, if there is a DSB occurring at a specific site in *wt* cells and the H3K4me3 mark is not already present due to transcriptional activity, Set1p is recruited to it in a RSC-dependent fashion (Figure 6). Either concomitant to this recruitment or thereafter, Set1p will induce *de novo* methylation of H3K4 on the nucleosomes surrounding the site (Figure 3). RSC, a SWI/SNF type chromatin remodelling complex, has recently been shown to associate with DSB very early and the preliminary time-coarse data reported here is consistent with those results ([62], [63], Figure 7). The results further suggest that this may be true for the occurrence of H3K4me3 too (Figure 7). Similar to what is shown here for the H3K4me3 modification, the recruitment of Mre11p, another protein involved in early recognition DNA damage, is also RSC-dependent [63]. In addition, cells lacking Rsc30p, a non-essential member of RSC, display a number of indistinguishable phenotypes as cells lacking Set1p: an important reduction of NHEJ repair and an increased sensitivity to chronic exposure to HO endonuclease (Figure 2, [13]) and a marked sensitivity to HU mediated S-phase arrest (Figure 5C). Consistent with a close relationship between RSC, Set1p and the H3K4me3 mark, the respective genes genetically appear to be in the same epistasis group in terms of sensitivity to genotoxic agents (Figure 6). We therefore speculate that nucleosome remodelling by the RSC-complex in conjunction with a relative opening of the chromatin by H3K4 methylation could be important for the ensuing DNA repair mechanisms. However, we failed to be able to demonstrate a direct physical interaction between H3K4me3 and the RSC catalytic subunit Sth1p by co-immunoprecipitation (data not shown).

The results also strongly suggest that an absence of H3K4me3 in cells with damaged DNA causes defects in traversing S-phase; presumably the problems occur when replication forks encounter the site of DNA damage (Figure 5). Recent evidence suggests that the MRX complex plays a variety of roles in damage recognition and checkpoint control during S-phase [64]. Interestingly, some of those MRX functions are independent of the S-phase checkpoint and our data here show that in the presence of DNA damage, cells lacking any of the MRX genes, but not those lacking *TEL1*, display a high degree of synthetic lethality with a *SET1* deletion (Figure 1). It is therefore plausible that MRX and *SET1* define two independent pathways that become important for passage through S-phase in the presence of damage, which would explain our synthetic interaction data (Figure 1, Figure 2, Figure 5). A very comparable genetic interaction between the MRX complex and *SET1* as the one reported here for budding yeast has also been found in *S. pombe* [65], which is consistent with the idea that this convergence of two pathways on the DSB repair process is conserved.

Set1p has also been shown to interact with and somehow modify the activity of Mec3p, a conserved checkpoint protein [31]. In addition, in mammalians, there is increasing evidence for an association of tumour suppressor genes with H3K4me3. For example, both the *ING1* and *ING4* proteins that are associated with DNA repair and cellular transformation bind to H3K4me3 [66], [67]. These findings hint to a widespread conservation of this alternate function of the H3K4me3 histone modification brought about by Set1p and homologous proteins in genome stability mechanisms.

This report highlights the complexity of downstream effects due to histone modifications and the occurrence of separate influences on major biological processes such as transcription, DNA replication and DNA damage repair. H3K4me3 generally is accepted as a very important mark in the genome of active promoters and the occurrence of recent transcription [45], [46], [68]. Here we report our discovery of another, most likely independent role for this chromatin modification: perhaps combined with the recruitment of chromatin remodeling activities it acts as a key player in the pathways governing genome stability in case of DNA damage.

## Materials and Methods

### Nomenclature

We use the proposed histone modification nomenclature [69]. Trimethylation of the histone H3 on the lysine residue 4 becomes H3K4me3.

### Strains and plasmids

All individual *S. cerevisiae* strains used in this study are listed in Table S1. All strains are isogenic with the S288c background; except strains JKM179, JKM181, YZS276, YZS246, DFY023, yFR016, DFY027, DFY028, DFY035, DFY036, DFY037, DFY046 and DFY047 that were derived from a W303 background. Plasmid YCpHOcut4 has been described before [70].

The strain used for SGA analysis was MLY532 (Matα *rad50*::*NatR can1*::MFA1pr-*HIS3*-MFalpha1pr-*LEU2 his3Δ leu2Δ0 ura3Δ0 met15Δ0 lys2Δ0*). To create this strain, Y3556 (Matα *can1*::MFA1pr-*HIS3*-MFalpha1pr-*LEU2 his3Δ leu2Δ0 ura3Δ0 met15Δ0 lys2Δ0)* [71] was transformed with a plasmid pJH1032-ad50D-NatR linearized by *ClaI*. pJH1032-rd50D-NatR was constructed using pJH1032 (former name pNKY1070 [72]) in which the *NatR* marker gene replaces a *Xba1* fragment of *RAD50*. MLY530 (Matα *mre11*::*NatR can1*::MFA1pr-*HIS3*-MFalpha1pr-*LEU2 his3Δ leu2Δ0 ura3Δ0 met15Δ0 lys2Δ0*) was created by transforming a *BamH1*+*Xho1* fragment of plasmid pRS313-MRE11d-NatR. To construct pRS313-MRE11d-NatR, *MRE1* coding sequences were amplified using oligos CTCGAAACTAGTGGATCTCAAACA and CTTGCTATACGAATTCAAGAGCAAAG harbouring respectively *SpeI* and *EcoRI* restriction sites. This fragment was then cloned into pRS313 using the added restriction sites. The resulting plasmid was digested with *HpaI* and we replaced the excised 2135 bp fragment with a blunted *NatR* fragment creating pRS313-MRE11d-NatR.

These strains were crossed with the library of nearly 4700 individual haploid strains with the a mating type (background strain BY4741) purchased from Invitrogen Life Technologies. Information is available at the Saccharomyces Deletion project Website. In order to confirm potential hits, we created diploid strains DFY001, DFY002, DFY004 by crossing haploid strains MLY530 and MLY532 with the respective strain from the yeast deletion library.

Strain DFY06 (*hht1*-*hhf1*Δ *hht2*-*hhf2*Δ *rad50*Δ::*NatR* pMS329 (*HHT1*-*HHF1 CEN URA3*) was derived from MSY421 (*hht1*-*hhf1*Δ *hht2*-*hhf2*Δ pMS329 (*HHT1*-*HHF1 CEN URA3*) [73]. DFY07 and DFY08 were obtained by a plasmid shuffle introducing plasmids encoding the wild type or mutated versions of histone H3: pZS136 (*HHT2*-*HHF2 CEN TRP1*), pZS138 (*hht2*-*K4R*-*HHF2 CEN TRP1*) and pZS138 (*hht2*-*K79R*-*HHF2 CEN TRP1*) [22], obtained from Brian Strahl, UNC-Chapel Hill) with strain DFY06. Transformation of plasmids and DNA into yeast was performed as described [74].

Mutant haploid strains were constructed by replacing the ORF of the targeted gene in parental strain LLY33 (for MT0-73, DFY003, DFY006, DFY011, DFY024, DFY049), YW1276 (DFY021, DFY022), JKM179 (for DFY023), yFR016 (DFY027, DFY028, DFY037), YZS267 (DFY038), DFY017 (DFY039), FT4 Sth1-9myc (DFY047) and a spore of DFY042 (DFY044) with the indicated selection markers using a PCR-mediated gene disruption method [75]. The LLY33 strain was created by micro-dissection of the diploid BY4705 [75]. Strains DFY008, DFY009 and DFY014 were constructed by the integration of a digested fragment derived from the *rad50*Δ::*NatR* plasmid into the genomic locus [76]. All strains used in this study had their mutations confirmed by marker segregation and PCR analysis. Strain DFY048 (13XMYC-YKU80) was constructed by adding a C-terminal myc epitope tag to Yku80p using a PCR-based method.

### General yeast growth conditions

Cells were grown on standard rich YEPD media containing, if needed, 100 ug/ml of nourseothricin (Clonat, WERNER Bioagents, Germany) or G418 at 150 ug/ml (Sigma). In some cases, synthetic medium YC supplemented with needed amino acids and bases was used. All genetic manipulations were performed as described previously [77], [78].

### Flow cytometry analysis

Cell synchronizations were performed as published with minor modifications [79], [80]. Following a three hours cell synchronization in G1 using α-factor, phleomycin was added to cultures to reach indicated final concentrations and cells were further incubated for 90 min. In order to remove the DNA damaging agent and the α-factor, cells were washed two times with sterile water and resuspended in fresh growth media. At the respective time points of the specific experiments, aliquots were processed for FACS analyses [81] or budded cells were counted using an hemacytometer and percentage of budded cells was calculated as the ratio of budded cells over all cells. For the S-phase release experiments, cells were synchronized in G1 phase for three hours as above, washed two times using sterile water in order to remove the pheromone and resuspended in YEPD media containing 200 mM Hydroxyurea (HU). Cells were left in this media for 60 min, washed again using sterile water and finally resuspended in fresh YEPD media containing Nocodazole for the rest of the experiment. Aliquots were again prepared for FACS analyses at the indicated time points.

### Drug sensitivity assays

Cell sensitivity to damaging agents was verified by spot tests on YEPD media containing the indicated concentrations of MMS (Sigma), HU (Sigma) and Bleomycin (BLENOXANE, Bistrol Meyers). Mid-log cultures were spotted in serial 10-fold dilutions and were allowed to grow for 3 to 4 days, before documentation. In later stages of the project, we used Phleomycin in place of Bleomycin as genotoxic agent. In control experiments 0,01 µg/mL of Phleomycin caused the same phenotypes as 1 mU of Bleomycin (data not shown) and the two can be used interchangeably.

For viability experiments after exposure to HU, cells were grown to log phase in YEPD at 30°C and then arrested in G1 with α-factor for three hours. Such synchronized cultures were then washed three times and released into S-phase by resuspension in YEPD to which HU was added to yield a final concentration of 1M. Aliquots of cells were taken after 1, 3 and 5 hours of incubation, cell density determined by counting and aliquots of 2×10^3^ cells were plated on YEPD plates. Viability is expressed as the percentage of colonies with respect to the counted cells.

For sensitivity to DNA breaks induced by the HO-endonuclease, JKM179 cells and derivative strains were grown to log phase in YEP-media supplemented with 2% raffinose. Cell concentration was adjusted to 1×10^7^ cells/ml in YEPGal media and cells were incubated for 2, 4 or 6 hrs. Cells were then counted and 2×10^3^ cells were plated on YEPD media and incubated at 30°C for 2 days. The survival rate of *wt* cells was determined by dividing the number of colonies obtained by the number of cells plated. All mutant survival rates were compared to the wild type rate which was set to 1.

### Plasmid religation assay

In general, the experiment was performed as described previously [40]. The centromeric plasmid pRS316 was digested with the *BamHI* restriction enzyme and then transformed into yeast cells. The transformation of undigested plasmid done in parallel was used to assess transformation efficiency of each sample. Efficiency of religation was determined by the number of colonies that were able to grow on a media selecting for the marker contained on the plasmid and was normalized by the transformation efficiency for each sample. All religation efficiencies in mutant cells were compared to wild type cells which were set to 100% and *yku70*Δ and *lig4*Δ cells were used as controls.

### HOSD(+1) NHEJ assay

Essentially, the experiments were done as previously described [41]. Wild type, control strains carrying deletions of genes known to be involved in NHEJ (*yku70*Δ and *lig4*Δ) and *set1*Δ cells were grown in Yc-Met media containing Raffinose as carbon source for 16 hours. For an observation of a chronic exposure to break induction, 4×10^4^ cells were incubated in media containing either glucose or galactose and their re-growth characteristics monitored over 48 hrs using an automatic plate incubator and reader (see [82], for details).

Error-prone/Error-Free NHEJ-ratio was obtained by growing cells for 2 days in Yc-Met+Ade +2% raffinose media, followed by a dilution into Yc-complete supplemented with 2% galactose. Cells were allowed to grow for 2 days and then plated at appropriate dilutions onto Yc-complete +2% glucose plates to obtain the total of viable cells, and onto Yc-Ade +2% glucose plates in order to reveal the +2 imprecise NHEJ events. Results were determined by dividing the number of colonies on Yc-Ade plates (imprecise NHEJ) by the total colonies on Yc-complete (all NHEJ events).

### Assay for efficiency of homologous recombination

Wild type and *set1*Δ cells harbouring the YcpHocut4 plasmid were grown for overnight in Yc-Ura media +2% raffinose and diluted to 1×10^7^ cells/mL. A first control aliquot (non treated) was removed and cells were re-suspended into Yc-Ura +2% galactose for 1 hour. After this incubation, the second aliquot (galactose) was removed. The remaining culture was washed 3 times in sterile water and resuspended in Yc-Ura +2% glucose. Aliquots were taken at hourly intervals (t = 1 to t = 4). DNA was extracted, quantified and QPCR was performed on all samples in order to analyze the production of the *MAT* **α** switching product. For both strains, results were normalized to non-treated samples.

### Western analyses

Midlog cell cultures were either exposed to 10 µg/ml of Phleomycin for one hour or left untreated and protein extracts were prepared using a modified TCA method [83]. Proteins were separated on an 8% and 15% acrylamid-bisacrylamid gels (ratio 29∶1) using standard techniques [84]. Gels were transferred onto a PVDF membrane and treated according to manufacturer's instructions (Perkin Elmer). Anti-H3K4me3 polyclonal antibody was purchased from Abcam (Ab8580) and used at a 1∶ 5000 dilution. H3K4me0 peptide (a kind gift of Alain Verreault) was used with Ab8580 in order to reduce non-specific binding of the antibody. Anti-H3K4me2 polyclonal antibody was obtained from V. Geli. Polyclonal anti Phospho-H2AS129 (Ab15083) was purchased from Abcam and used at a 1∶750 dilution. Signals were detected using horseradish-peroxidase-coupled anti-rabbit secondary antibodies (GE Healthcare) and enhanced chemiluminescence (Perkin Elmer). Monoclonal anti-Pgk1p was obtained from Molecular Probes and used at a 1/20000 dilution.

### Northern analyses

RNA extraction and Northern blots were performed as previously described [85]. Cln2 and Hta1 RNAs were detected using radiolabeled probes prepared by a random priming method using the following primers:

Cln2 = TAACAGCAATAACGCAACCA and CCGCAACGGCGCATTACCT

Hta1 = ATGTCCGGTGGTAAAGGTGG and TCTTGAGAAGCCTTGGTAGC

Act1 = TCCGGTGATGGTGTTACTCA and ATTCTCAAAATGGCGTGAGG

Mata1 = CACCGCACAATTCATCATTTGCGT and CTGGGTAGAGTCTTATTGGCAAGA

### RT–QPCR analyses

Synthesis of cDNA was performed using 2 µg of total RNA and SuperScript II Reverse Transcriptase (Invitrogen, Burlington, ON). A gene-specific primer protocol using a “Flap sequence” was used to avoid DNA amplification during subsequent PCR amplification. RNase treated samples were used as negative controls. The cDNA was purified using the QIAgen mini-elute system (Qiagen Inc., Mississauga, ON, Canada). All primers used for RT PCR and the following QPCR are listed in Table S2.

### Chromatin ImmunoPrecipitations (ChIP)

DFY027 or DFY028 cells were arrested in G1 phase with α-factor for three hours in Yc-Ura +2% raffinose and the expression of the HO- endonuclease was induced or re-repressed by the addition of 2% galactose or 2% glucose, respectively. Chromatin samples were prepared essentially as described [86]. Slight modifications include: following formaldehyde-mediated crosslinking, cells were resuspended in 4 ml of ChIP buffer [86] and dropped into liquid nitrogen in order to form “popcorn-like” particles. Such frozen cell pellets were then lysed using a SPEX CertiPrep 6850 Freezer Mill to produce deep frozen powdered whole cell extracts. Extracts were thawed on ice and sonicated (30×15 pulses, setting 2, 40% output power) using a Branson Sonifier. H3K4me3 and myc immunoprecipitation were performed using respectively anti-H3K4me3 polyclonal antibody Abcam (Ab8580) and anti-myc antibody Roche (Clone 9E10). Detection of the indicated loci was achieved by PCR using the following primers:

HO cleavage site at mating type = ATTCTTAGCATCATTCTTTGTTC and TCCAATCTGTGCACAATGAAG;

*CENIV* = ATGTTGAAGGAACAGCTGGG and AGGCTCAATGTTGACTAGCC;

*PYK1* = GAAACGATAAGTGCTACTCCGTCCTA and GGTCATCTATGGGGCTTGAATCT.

### Time course HO-cleavage analysis and cleavage efficiency

Cells were grown overnight in Yc-Uracil +2% glycerol lactate medium. Once exponential phase reached, 2% galactose was added and aliquots taken at the specified time points. Cells were then prepared for ChIP analysis or DNA was directly extracted for cleavage efficiency. HO cleavage efficiency was calculated by QPCR using primers listed in Table S2. These primers overlap the cleavage site. Percentage of cleavage was calculated by dividing the number of QPCR amplicons of experimental samples by the number obtained in non-induced cells. Cells grown in glucose (non-induced) were set at a cleavage efficiency of 0%.

### Quantitative PCR analyses

For QPCR analyses, specific sequences were amplified by real-time PCR using either a Rotor-Gene RG-3000A or a Rotor-Gene RG-3000 (Corbett Research) and the FastStart SYBR Green Master kit (Roche Applied Sciences). The different primers used are listed in Table S2. Each 10 µl reaction containing 2 µl of DNA sample and 0.3 µM of primers was quantified in triplicate 30 to 45 cycles of 15 s at 94°C, 30 s at 53° to 59°C (Table S2) and 30 s at 72°C.

## Supporting Information

Figure S1 Genetic interaction between *BRE2* and the MRX complex. Sensitivity to the radiomimetic drug Bleomycin of strains harbouring deletions of genes encoding COMPASS complex subunits, *BRE2* in (A) and *SPP1* in (B) in combination with a deletion of *RAD50*. Serial ten-fold dilution growth tests of exponentially growing cultures on plates without Bleomycin (YEPD control, left) and with Bleomycin (right) are shown. (C) Western blot analysis of H3K4me2 and H3K4me3 levels in *wt* (LLY33) *set1*Δ (DFY011) *bre2*Δ (BY4741 YLR015W) and *spp1*Δ (BY4741 YPL138C) cells. Pgk1p was used as a loading control.
(1.43 MB TIF)

Figure S2 Levels of precise or imprecise NHEJ in *set1.* (Δ. A) Schematic of the gal-HO region flanked by two HO-recognition sites in strain YW1276. Upon cleavage of the indicated sites, NHEJ can occur in a precise fashion which will not recreate the *ADE2* ORF, resulting in red colonies or the *ADE2* orf can be reconstituted by imprecise (+2) NHEJ, yielding white colonies. (B) Overall percentages of precise and imprecise NHEJ as determined in wild type cells *wt* (YW1276), *set1*Δ (DFY021), *yku70*Δ (YW1283) or *dnl4*Δ (DFY022) cells. Note that total surviving colonies (precise plus imprecise NHEJ) in wild-type cells was set to 100% and the fraction of colonies of the respective mutant cells were expressed in relation to that. (C) Genomic HO cleavage efficiency in both *wt* (YW1276) and *set1*Δ (DFY021) cells grown in galactose for 1 hour. (D) HO cleavage efficiency of the cleavage site on plasmid YcpHocut4 of *wt* (DFY027) and *bre1*Δ (DFY028) cells incubated in galactose for the indicated number of hours. Genomic locus hybridization serves as a DNA loading control.
(1.11 MB TIF)

Figure S3 Normal levels of homologous recombination in *set1*Δ cells. Analysis of the mating type switching of strains *wt* (DFY046) and *set1*Δ (DFY047). Strains were initially *MAT*a and switched to *MAT*α following induction of the HO cleavage at mating type locus. Appearance of *MAT*α switching product was measured by QPCR using oligos specific for *MAT*α locus. Values were normalized with the amount of switching product found in non-induced samples (glucose) which was set to 1.
(0.70 MB TIF)

Figure S4 Variation of histone modification levels after treatment of cells with Phleomycin. Wild type cells ÿLLY33) were exposed to 1, 10 or 100 µg/mL of phleomycin and levels of H3K4me3 and H2AS129P (yeast γ-H2A) were assessed by western blotting. *set1*Δ (DFY021) cells were used as a negative control for H3K4me3 protein modification and Pgk1p was used as a loading control.
(0.75 MB TIF)

Figure S5 Mata1 RNA levels do not vary upon treatment of cells with galactose. (A) Northern blot analysis of Mata1 RNA derived from *wt* (DFY027) cells that contain a gal-HO-endonuclease gene on a plasmid and that were grown in glucose or galactose media as indicated. Signals for Mata1 RNA were quantified using a Phosphorimager (Storm) and corrected for loading with using the signals for Act1 mRNA (below). Numbers below each lane indicate the actual ratio if the first lane was set to 1. (B) The same RNA samples as in (A) were used and relative levels quantified by RT-QPCR. RNase treated samples served negative controls. (C) Localization of H3K4me3 to the *MAT* locus in cells harbouring a *MAT*a-inc allele and where the HO site is not cleaved. Cells harbor an integrated copy of the gal-HO gene and were grown either in glucose or in galactose as indicated.
(0.82 MB TIF)

Figure S6 Lack of NHEJ components does not sensitize cells to hydroxyurea. Serial ten-fold dilution growth tests of exponentially growing cultures of *wt* (JKM179), *set1*Δ (DFY023), *yku70*Δ (JKM181), *rad52*Δ (MT0-73) cells on plates with 50 mM hydroxyurea. Colonies were allowed to grow for three days at 30°C.
(0.95 MB TIF)

Table S1 Strains used in this study.
(0.43 MB RTF)

Table S2 Oligonucleotides used in this study.
(0.06 MB RTF)
