# Supplementary figures and images for: Methylated H3K4, a Transcription-Associated Histone Modification, Is Involved in the DNA Damage Response Pathway
Source: PLoS Genet. 2010 Aug 26;6(8):e1001082. doi: 10.1371/journal.pgen.1001082 (PMC2928815; doi:10.1371/journal.pgen.1001082)

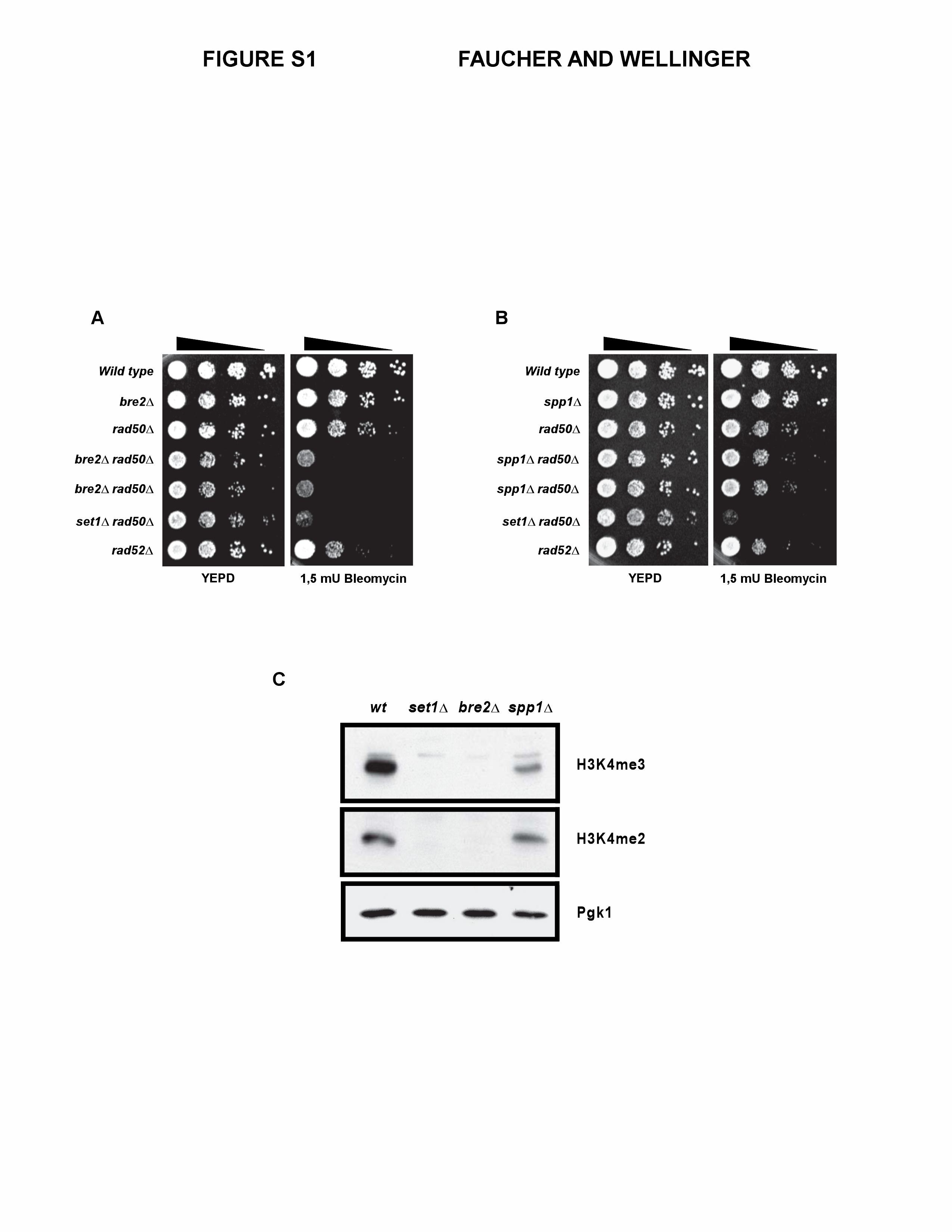

Supplement: Figure S1 — Genetic interaction between BRE2 and the MRX complex. Sensitivity to the radiomimetic drug Bleomycin of strains harbouring deletions of genes encoding COMPASS complex subunits, BRE2 in (A) and SPP1 in (B) in combination with a deletion of RAD50. Serial ten-fold dilution growth tests of exponentially growing cultures on plates without Bleomycin (YEPD control, left) and with Bleomycin (right) are shown. (C) Western blot analysis of H3K4me2 and H3K4me3 levels in wt (LLY33) set1Δ (DFY011) bre2Δ (BY4741 YLR015W) and spp1Δ (BY4741 YPL138C) cells. Pgk1p was used as a loading control. (1.43 MB TIF) [file pgen.1001082.s001.tif]

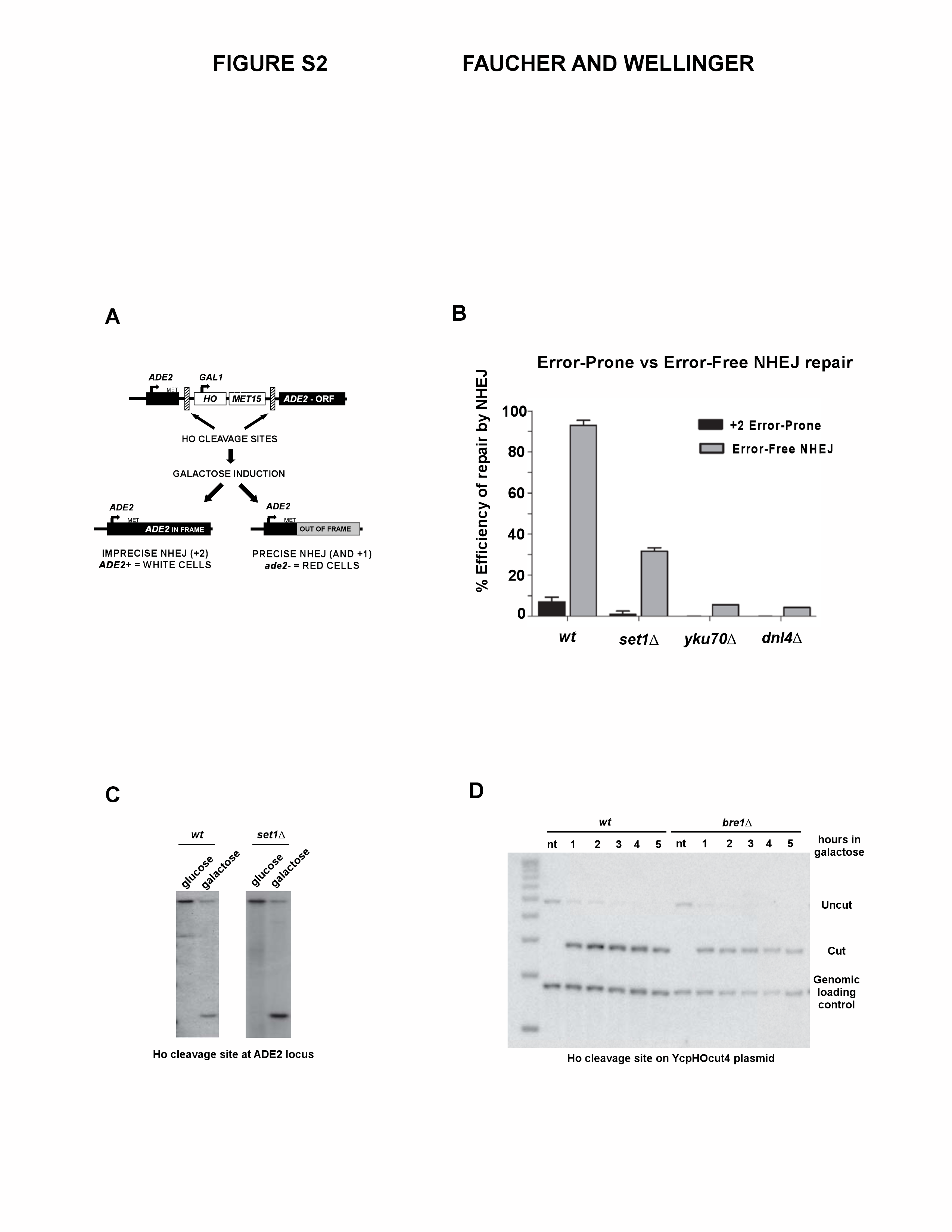

Supplement: Figure S2 — Levels of precise or imprecise NHEJ in set1. (Δ. A) Schematic of the gal-HO region flanked by two HO-recognition sites in strain YW1276. Upon cleavage of the indicated sites, NHEJ can occur in a precise fashion which will not recreate the ADE2 ORF, resulting in red colonies or the ADE2 orf can be reconstituted by imprecise (+2) NHEJ, yielding white colonies. (B) Overall percentages of precise and imprecise NHEJ as determined in wild type cells wt (YW1276), set1Δ (DFY021), yku70Δ (YW1283) or dnl4Δ (DFY022) cells. Note that total surviving colonies (precise plus imprecise NHEJ) in wild-type cells was set to 100% and the fraction of colonies of the respective mutant cells were expressed in relation to that. (C) Genomic HO cleavage efficiency in both wt (YW1276) and set1Δ (DFY021) cells grown in galactose for 1 hour. (D) HO cleavage efficiency of the cleavage site on plasmid YcpHocut4 of wt (DFY027) and bre1Δ (DFY028) cells incubated in galactose for the indicated number of hours. Genomic locus hybridization serves as a DNA loading control. (1.11 MB TIF) [file pgen.1001082.s002.tif]

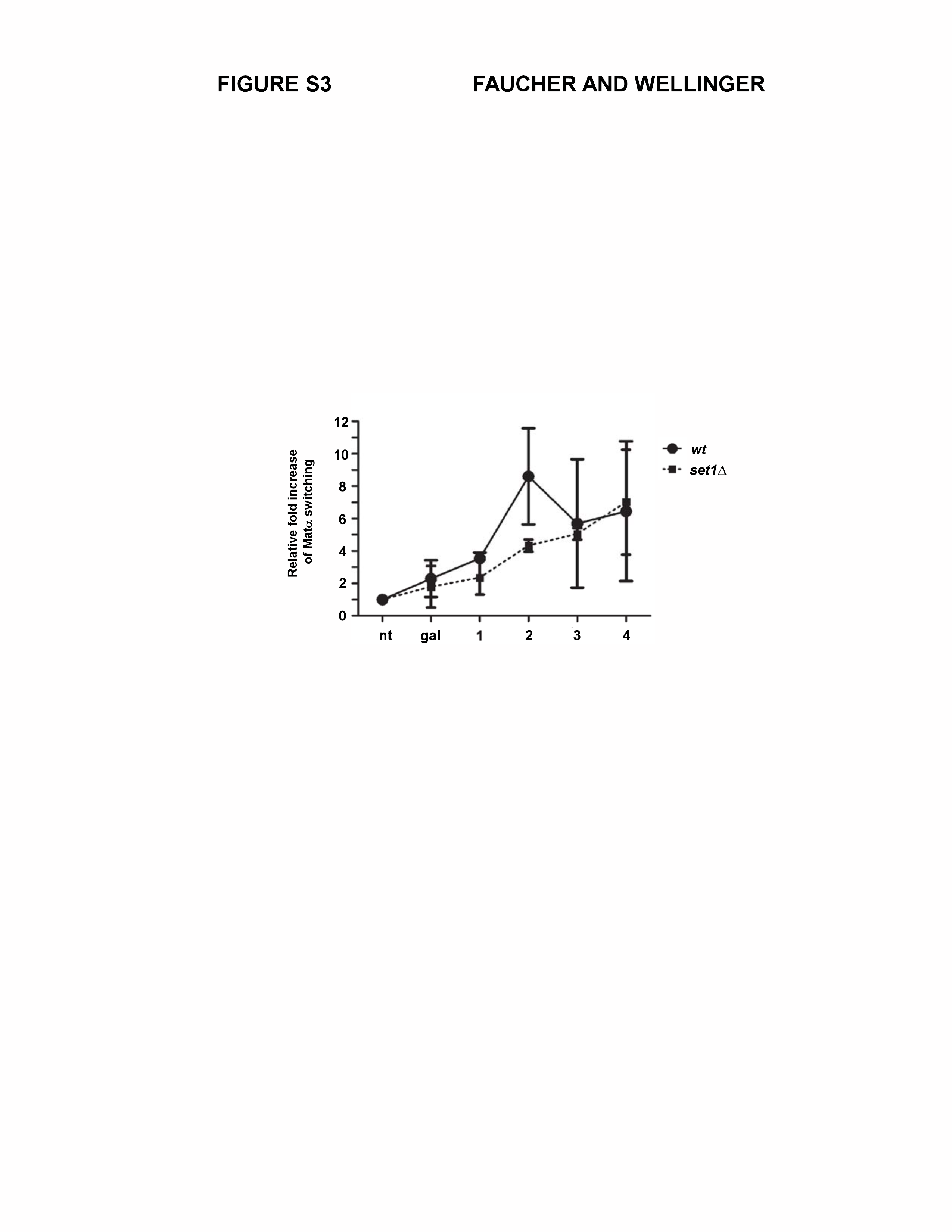

Supplement: Figure S3 — Normal levels of homologous recombination in set1Δ cells. Analysis of the mating type switching of strains wt (DFY046) and set1Δ (DFY047). Strains were initially MATa and switched to MATα following induction of the HO cleavage at mating type locus. Appearance of MATα switching product was measured by QPCR using oligos specific for MATα locus. Values were normalized with the amount of switching product found in non-induced samples (glucose) which was set to 1. (0.70 MB TIF) [file pgen.1001082.s003.tif]

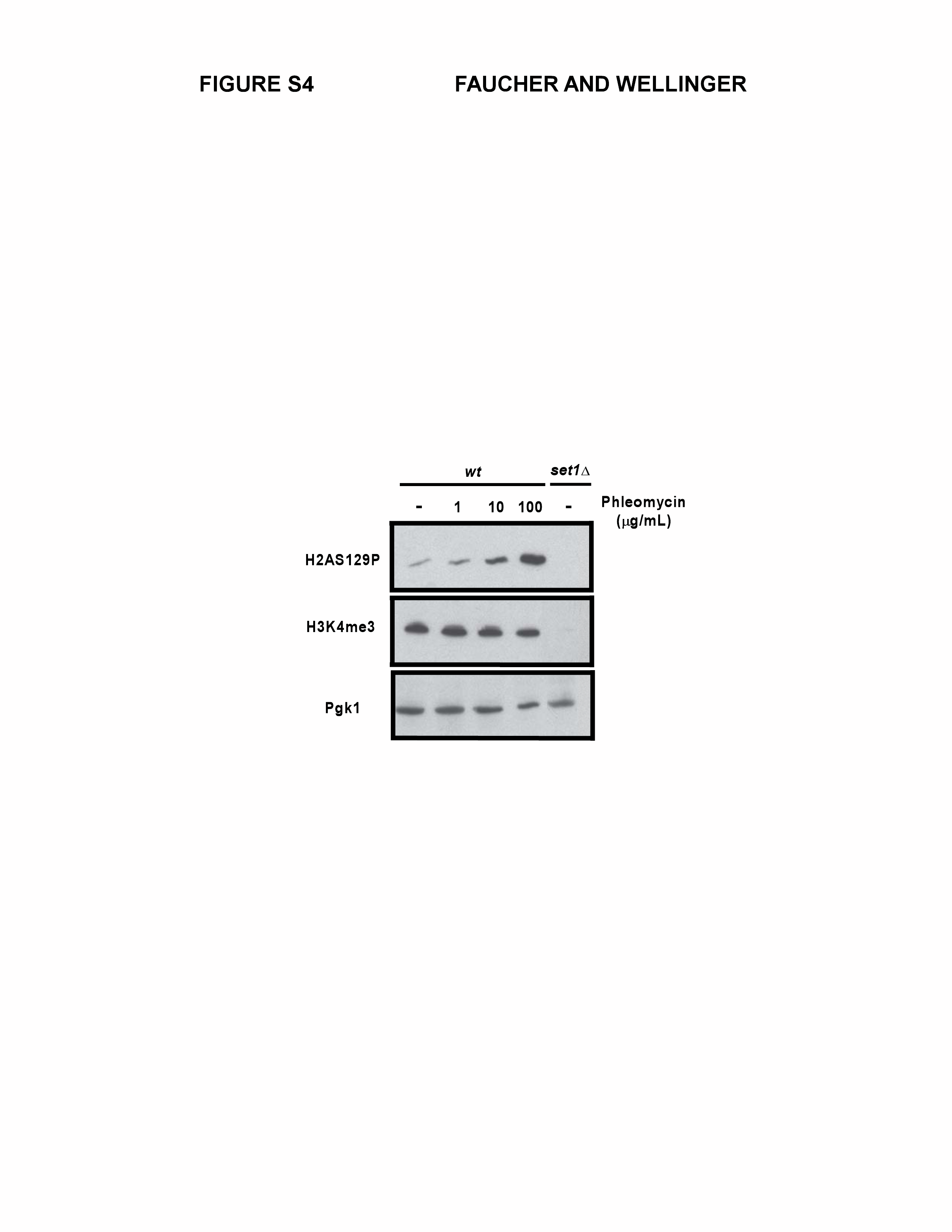

Supplement: Figure S4 — Variation of histone modification levels after treatment of cells with Phleomycin. Wild type cells ÿLLY33) were exposed to 1, 10 or 100 µg/mL of phleomycin and levels of H3K4me3 and H2AS129P (yeast γ-H2A) were assessed by western blotting. set1Δ (DFY021) cells were used as a negative control for H3K4me3 protein modification and Pgk1p was used as a loading control. (0.75 MB TIF) [file pgen.1001082.s004.tif]

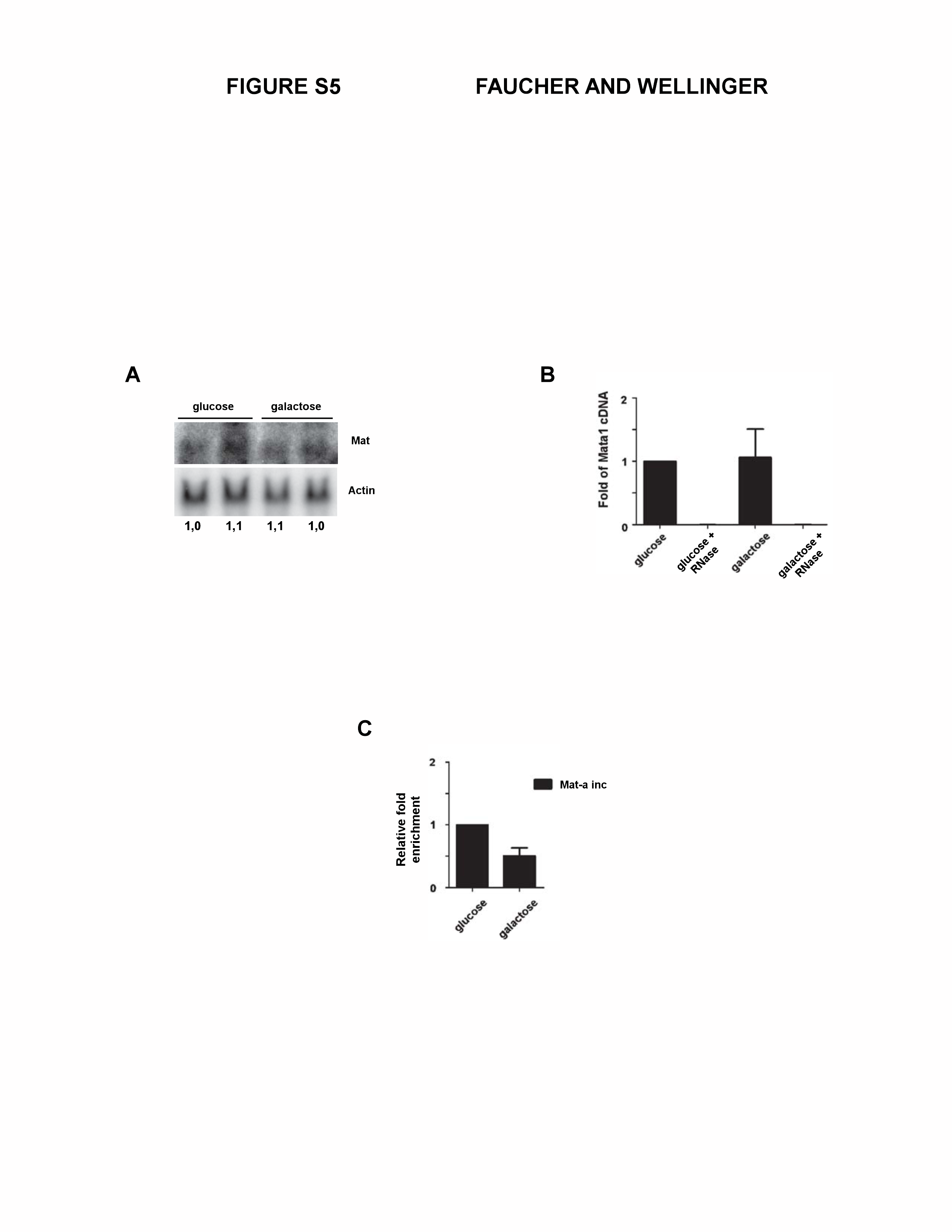

Supplement: Figure S5 — Mata1 RNA levels do not vary upon treatment of cells with galactose. (A) Northern blot analysis of Mata1 RNA derived from wt (DFY027) cells that contain a gal-HO-endonuclease gene on a plasmid and that were grown in glucose or galactose media as indicated. Signals for Mata1 RNA were quantified using a Phosphorimager (Storm) and corrected for loading with using the signals for Act1 mRNA (below). Numbers below each lane indicate the actual ratio if the first lane was set to 1. (B) The same RNA samples as in (A) were used and relative levels quantified by RT-QPCR. RNase treated samples served negative controls. (C) Localization of H3K4me3 to the MAT locus in cells harbouring a MATa-inc allele and where the HO site is not cleaved. Cells harbor an integrated copy of the gal-HO gene and were grown either in glucose or in galactose as indicated. (0.82 MB TIF) [file pgen.1001082.s005.tif]

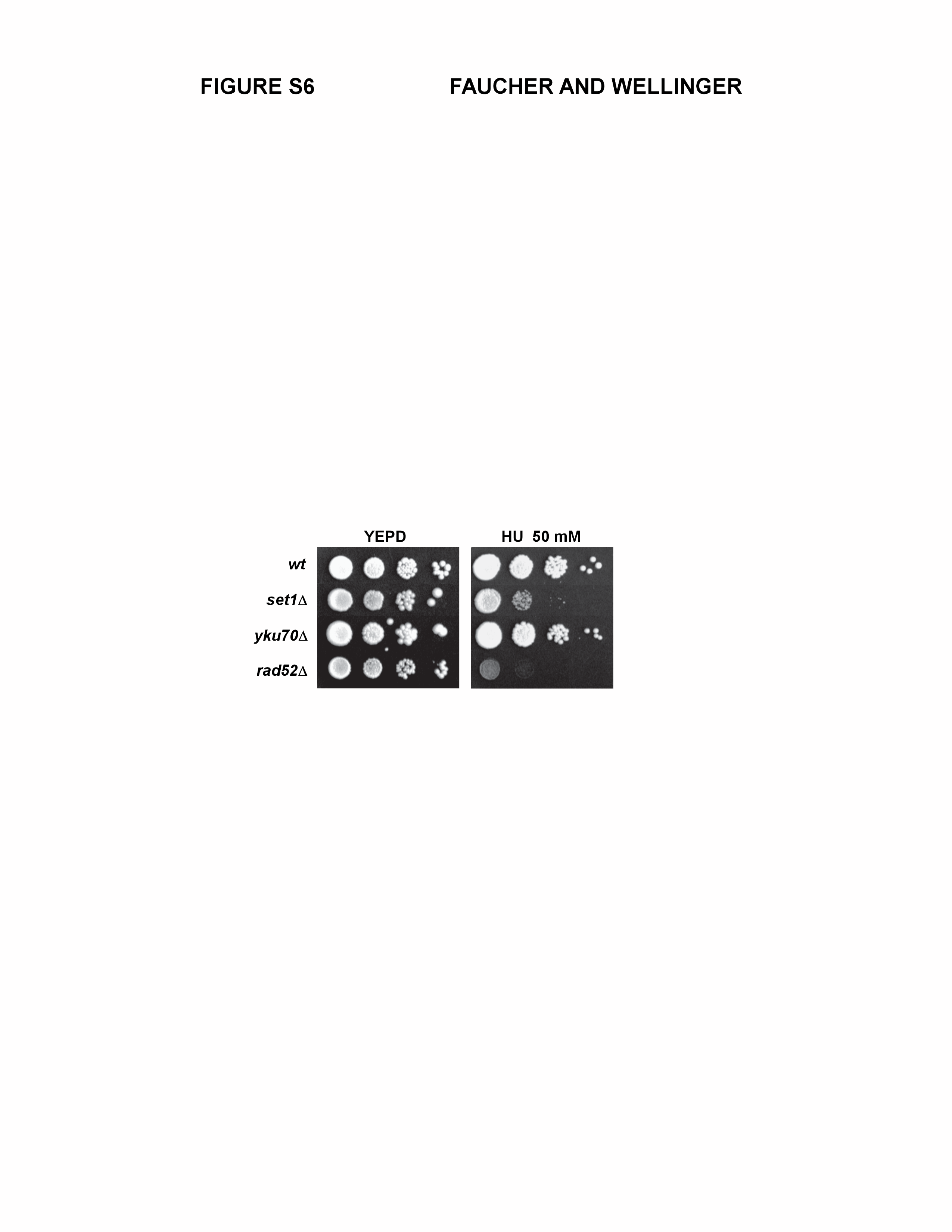

Supplement: Figure S6 — Lack of NHEJ components does not sensitize cells to hydroxyurea. Serial ten-fold dilution growth tests of exponentially growing cultures of wt (JKM179), set1Δ (DFY023), yku70Δ (JKM181), rad52Δ (MT0-73) cells on plates with 50 mM hydroxyurea. Colonies were allowed to grow for three days at 30°C. (0.95 MB TIF) [file pgen.1001082.s006.tif]
