# Supplementary material for: Methylated H3K4, a Transcription-Associated Histone Modification, Is Involved in the DNA Damage Response Pathway
Source: PLoS Genet. 2010 Aug 26;6(8):e1001082. doi: 10.1371/journal.pgen.1001082 (PMC2928815; doi:10.1371/journal.pgen.1001082)
Supplement: Table S1 — Strains used in this study. (0.43 MB RTF) [file pgen.1001082.s007.rtf]

Strain	Genotype	Reference	
Y3656	can1Ä::MFA1pr-HIS3-Mha1pr-LEU2   his3Ä1   leu2Ä0   met15Ä0 ura3Ä0	Tong et al. 2001	
MLY532	MATá   rad50Ä::NatR  can1Ä::MFA1pr-HIS3-Mha1pr-LEU2 his3Ä1   leu2Ä0   met15Ä0   ura3Ä0	This study	
MLY530	MATá   his3Ä1   leu2Ä0   met15Ä0   ura3Ä0   mre11Ä::NatR  can1Ä::MFA1pr-HIS3-Mha1pr-LEU2	This study	
BY4741	MATa   his3D1   leu2D0   met15D0   ura3D0	INVITROGEN	
BY4705	MATa/MATá    ade2ÄhisG/ADE2   his3Ä200/his3Ä200  leu2Ä0/leu2Ä0   lys2Ä0/lys2Ä0   met15Ä0/MET15   trp1Ä63/TRP1    ura3Ä0/ura3Ä0	Brachmann et al. 1998	
BY4741
YPL055C	MATa   his3D1   leu2D0   met15D0   ura3D0   lge1D::KmX	INVITROGEN
	
BY4741
YDL074C	MATa   his3D1   leu2D0   met15D0   ura3D0   bre1D::KmX	INVITROGEN
	
BY4741
YHR119W	MATa   his3D1   leu2D0   met15D0   ura3D0   set1D::KmX	INVITROGEN
	
BY4741
YDR440W	MATa   his3D1   leu2D0   met15D0   ura3D0   dot1D::KmX	INVITROGEN
	
BY4741
YHR056W	MATa   his3D1   leu2D0   met15D0   ura3D0   rsc30D::KmX	INVITROGEN
	
BY4741
YOR141C	MATa   his3D1   leu2D0   met15D0   ura3D0   arp8D::KmX	INVITROGEN
	
BY4741
YCL060C	MATa   his3D1   leu2D0   met15D0   ura3D0   mrc1D::KmX	INVITROGEN
	
BY4741
YOR064C	MATa   his3D1   leu2D0   met15D0   ura3D0   yng1D::KmX	INVITROGEN
	
BY4741
YGR056W	MATa   his3D1   leu2D0   met15D0   ura3D0   rsc1D::KmX	INVITROGEN
	
BY4741
YLR015W	MATa   his3D1   leu2D0   met15D0   ura3D0   bre2D::KmX	INVITROGEN
	
BY4741
YPL138C	MATa   his3D1   leu2D0   met15D0   ura3D0   spp1D::KmX	INVITROGEN
	
DFY001	MATa/MATá   met15Ä0/met15Ä0  ura3Ä0/ura3Ä0  rad50Ä::NatR/+   +/bre1Ä::KmX	This study	
DFY002	MATa/MATá   met15Ä0/met15Ä0  ura3Ä0/ura3Ä0  rad50Ä::NatR/+   +/lge1Ä::KmX   	This study	
DFY003	met15Ä0  ura3Ä0   rad50Ä::NatR   lge1Ä::KmX   	This study	
DFY004	MATa/MATá  met15Ä0/met15Ä0  ura3Ä0/ura3Ä0  mre11Ä::NatR/+   +/bre1Ä::KmX   	This study	
MCY751-2C	ade2ÄhisG   his3Ä200   leu2Ä0   lys2Ä0   ura3Ä0   met15Ä0  trp1Ä63   tel1Ä::LEU2	Chakhparonian et al. 2005	
DFY005	MATa/MATá   tel1Ä::LEU2/+   bre1Ä::KmX/+   ade2ÄhisG/+   his3Ä1/ his3Ä2000   leu2Ä0/leu2Ä0   met15Ä0/met15Ä0   ura3Ä0/ura3Ä0	This study	
LLY33	MATa   his3Ä200   leu2Ä0   lys2Ä0   ura3Ä0	This study	
DFY006	MATa   his3Ä200   lys2Ä0   ura3Ä0   bre1DLEU2	This study	
DFY007	MATa/MATá   met15Ä0/+  lys2Ä0/+  ura3Ä0/ura3Ä0   rad50Ä::NatR/+   +/bre1Ä:: LEU2	This study	
MT0-73	  MATa   his3D1   leu2D0   met15D0   ura3D0   rad52Ä::LEU2   	Toussaint et al. 2006	
YZS276	MATa (hta1-htb1Ä::LEU2 hta2-htb2Ä::LEU2 
+ pZS145(HTA1-FLAG-HTB1 CEN HIS3)	Sun and Allis 2002	
DFY008	MATa   (hta1-htb1Ä::LEU2 hta2-htb2Ä::LEU2 rad50Ä::NatR 
+ pZS145(HTA1-FLAG-HTB1 CEN HIS3)	This study	
YZS246	MATa   (hta1-htb1Ä::LEU2 hta2-htb2Ä::LEU2 
+ pZS140(HTA1-FLAG-htb1-K123R 2ì HIS3)	Sun and Allis 2002	
DFY009	MATa   (hta1-htb1Ä::LEU2 hta2-htb2Ä::LEU2 rad50Ä::NatR 
+ pZS140(HTA1-FLAG-htb1-K123R 2ì HIS3)	This study	
DFY010	MATa/MATá   met15Ä0/+   lys2Ä0/+   ura3Ä0/ura3Ä0   rad50Ä::NatR/+   dot1Ä::KmX/+   	This study	
DFY011	MATa   his3Ä200   lys2Ä0   leu2Ä0   set1D::URA3	This study	
DFY012	MATa/MATá   met15Ä0/+   lys2Ä0/+   ura3Ä0/ura3Ä0   rad50Ä::NatR/+   set1D::URA3/+	This study	
MSY421	MATa   (hht1-hhf1Ä hht2-hhf2Ä )
+ pMS329(HHT1-HHF1 CEN URA3)	Sun and Allis 2002	
YZS267	MATa (hht1-hhf1Ä hht2-hhf2Ä) 
+ pZS136(HHT1-HHF1 CEN TRP1)	Sun and Allis 2002	
DFY013	MATa   (hht1-hhf1Ä hht2-hhf2Ä) 
+ p[hht2-K79R-HHF2 CEN TRP1] 	Brian Strahl UNC	
DFY014	MATa   (hht1-hhf1Ä hht2-hhf2Ä ) rad50Ä::NatR 
+ pMS329(HHT1-HHF1 CEN URA3)	This study	
DFY015	MATa   (hht1-hhf1Ä hht2-hhf2Ä ) rad50Ä::NatR 
+ pMS329(HHT1-HHF1 CEN TRP1)	This study	
DFY016	MATa   (hht1-hhf1Ä hht2-hhf2Ä) rad50Ä::NatR  
+ p[hht2-K79R-HHF2 CEN TRP1]	This study	
DFY017	  MATa   (hht1-hhf1Ä hht2-hhf2Ä) 
+ pZS138(hht2-K4R-HHF2 CEN TRP1)	This study	
DFY018	MATa   (hht1-hhf1Ä hht2-hhf2Ä) rad50Ä::NatR 
+ pZS138(hht2-K4R-HHF2 CEN TRP1)	This study	
DFY019	MATa/MATá   his3Ä1/his3Ä1   lys2Ä0/+  ura3D0/ura3D0      met15Ä0/+  leu2D0/leu2D0   rad52Ä::LEU2/+  
  set1D::URA3/+	This study	
DFY020	MATa/MATá   his3Ä1/his3Ä1   lys2Ä0/+   ura3D0/ura3D0  met15Ä0/+   leu2D0/leu2D0   rad52Ä::LEU2/+   set1D::URA3/+    +/dnl4D::KmX	This study	
YW1276	MATá-inc  ade2::HOSD(+1)::STE3-MET15   his3Ä1   leu2D0 met15Ä0   ura3D0      	Della et al. 2004	
YW1283	YW1276   yku70D::HIS3	Della et al. 2004	
DFY021	YW1276   set1D::KmX	This study	
DFY022	YW1276   dnl4D::KmX  	This study	
JKM179	hoÄ MATá   hmlÄ::ADE1   hmrÄ::ADE1   ade1-100   leu2-3,112 trp1::hisG'   lys5   ura3-52   ade3::GAL::HO	Lee et al. 1999	
JKM181	JKM179   yku70D::URA3	Lee et al. 2003	
DFY023	JKM179   set1D::KmX	This study	
DFY024	MATa   his3Ä200   lys2Ä0   ura3Ä0   bar1D::HIS3	This study	
DFY025	MATa/MATá   his3Ä200/his3Ä200   lys2Ä0/ lys2Ä0   leu2Ä0/leu2Ä0  +/bar1DHIS3   +/set1D::URA3	This study	
DFY026	MATa   his3Ä200   lys2Ä0   leu2Ä0   bar1D::HIS3   set1D::URA3	This study	
yFR016	MATa   ura3-52   trp1-Ä63   his3-Ä200   leu2::PET56   3XHA-SET1 (Also named  FT4  3XHA-SET1)	Ng et al. 2002	
DFY027	MATa   ura3-52   trp1-Ä63   his3-Ä200   leu2::PET56   3XHA-SET1   bar1D::HIS3   +   YcpHocut4	This study
	
DFY028	MATa   ura3-52   trp1-Ä63   his3-Ä200   leu2::PET56   3XHA-SET1   bar1D::HIS3   bre1D::KmX   +   YcpHocut4	This study
	
DFY029	MATa  his3Ä200  lys2Ä0   ura3Ä0   bar1D::HIS3   rsc30D::KmX	This study	
DFY031	MATa  his3Ä200  lys2Ä0   ura3Ä0   bar1D::HIS3   arp8D::KmX	This study	
DFY032	MATa/MATá   his3Ä200/his3Ä200   lys2Ä0/ lys2Ä0   leu2Ä0/leu2Ä0  mrc1D::KmX/+   set1D::URA3/+   rad52Ä::LEU2/+	This study	
DFY033	MATa/MATá   met15Ä0/met15Ä0  ura3Ä0/ura3Ä0  rad50Ä::NatR/+ yng1D::KmX/+	This study	
DFY034	MATa/MATá   his3Ä200/his3Ä200   lys2Ä0/ lys2Ä0   leu2Ä0/leu2Ä0  bar1D::HIS3/+   set1D::LEU2/+   rad50Ä::NatR/+   rsc30D::KmX/+	This study	
FT4
Sth1-9myc	MATa ura3-52 trp1- Ä63 his3- Ä200 leu2::PET56 9XMYC-STH1 (Also named  FT4  9XMYC-STH1)	Ng et al. 2002	
DFY046	FT4  9XMYC-STH1 + YcpHocut4	This study	
DFY047	FT4  9XMYC-STH1  set1D::KmX + YcpHocut4	This study	
DFY035	FT4  9XMYC-STH1bar1D::HIS3   	This study	
DFY036	FT4  9XMYC-STH1 bar1D::HIS3   set1D::KmX + YcpHocut4	This study	
DFY037	MATa   ura3-52   trp1-Ä63   his3-Ä200   leu2::PET56   3XHA-SET1   bar1D::HIS3   rsc30D:: KmX   +   YcpHocut4	This study	
DFY038	MATa (hht1-hhf1Ä hht2-hhf2Ä) set1D::KmX  + pZS136(HHT1-HHF1 CEN TRP1)	This study	
 DFY039	MATa   (hht1-hhf1Ä hht2-hhf2Ä) set1D::KmX  + pZS138(hht2-K4R-HHF2 CEN TRP1)	This study	
MCY742	MATa/MATá   ade2::hisG / ade2   his3200 / his3   leu20 / leu2   lys20 / lys2   met150 / met15   trp163 / trp1   ura30 / ura3   mec1::mec1ts::HIS3 / mec1::TRP1   sml1::TRP1 / sml1::HIS3  TEL1 / tel1-11::natR   sae2::kanMX4 / SAE2	Chakhparonian et al., 2005	
DFY040	MATa/MATá   +/bre1ÄKmx +/mec1ÄTRP +/sml1ÄHIS  his3/his3  leu2/leu2  ura3/ura3	This study	
DFY041	bre1ÄKmx mec1ÄTRP sml1ÄHIS  his3  leu2  ura3	This study	
DFY042	MATa/MATá   +/lge1ÄKmx +/mec1ÄTRP +/sml1ÄHIS  his3/his3  leu2/leu2  ura3/ura3	This study	
DFY043	lge1ÄKmx mec1ÄTRP sml1ÄHIS  his3  leu2  ura3	This study	
DFY044	mec1ÄTRP sml1ÄHIS  bar1ÄKmx his3  leu2  ura3 	This study	
DFY045	MATa/MATá  met15Ä0/met15Ä0  ura3Ä0/ura3Ä0  mre11Ä::NatR/+   +/lge1Ä::KmX   	This study	
DFY048	LLY33  13XMYC-YKU80	This study	
DFY049	LLY33 set1D::LEU2  13XMYC-YKU80	This study	
DFY050	MATá/MATa   rad50Ä::NatR  can1Ä::MFA1pr-HIS3-Mha1pr-LEU2    his3Ä1/ his3Ä1   leu2Ä0/ leu2Ä0/  met15Ä0/ met15Ä0   ura3Ä0/ ura3Ä0  rsc1D::KmX/+	This study	
JHD1	Mata-inc/Matá hmlÄ::ADE1/hmlÄ::ADE1 hmrÄ::ADE1/hmrÄ::ADE1 HOÄ/HOÄ ade1-100/ade1-100 leu2-3,112/leu2-3,112 trp1::hisG/trp1::hisG lys5/lys5 ura3-52/ura3-52 ade3::GAL::HO/ade3::GAL::HO	J. Haber unpublished	
DFY051	Mata-inc hmlÄ::ADE1 hmrÄ::ADE1 HOÄ ade1-100 leu2-3,112 trp1::hisG lys5 ura3-52 ade3::GAL::HO	This study	
DFY052	Mata-inc hmlÄ::ADE1 hmrÄ::ADE1 HOÄ ade1-100 leu2-3,112 trp1::hisG lys5 ura3-52 ade3::GAL::HO + YcpHocut4	This study	
