# Supplementary material for: Methylated H3K4, a Transcription-Associated Histone Modification, Is Involved in the DNA Damage Response Pathway
Source: PLoS Genet. 2010 Aug 26;6(8):e1001082. doi: 10.1371/journal.pgen.1001082 (PMC2928815; doi:10.1371/journal.pgen.1001082)
Supplement: Table S2 — Oligonucleotides used in this study. (0.06 MB RTF) [file pgen.1001082.s008.rtf]

Oligo	Temperature of annealing (˚C)	Forward primer sequences	Reverse primer sequences	
MAT QPCR #2562 and #2777	59	5'- AGAATGGCACGCGGACAAAATG -3'	5'- CCACGACCACACTCTATAAGGCCAAA-3'	
MAT Cleavage QPCR 
#2397 and #2584	59	5'- TCACAGGATAGCGTCTGGAAGTCA -3'	5'-CATTTTGTCCGCGTGCCATTCT -3'	
MAT-HR-á QPCR	53	5'- AGTCACATCAAGATCGTTTATGG -3'	5'- GCACGGAATATGGGACTACTTCG-3'	
MAT RT
P0	-	5'- GATCATCACGGTGCCGGATCCCCCTTTGGGCTCTTCTCTTT -3'	-	
MAT RT QPCR
P1-P2	51	5'- GGCGGAAAACATAAACAGAA -3'	5'- GATCATCACGGTGCCGGATCC-3'	
Pho5-H QPCR	57	5'- TCATGTCCTGCTTGGGACTACGAT -3'	5'- CGTCAGTTGAGGTCAAGTTCAAACCC  -3'	
PYK1 QPCR	59	5'- GAAACGATAAGTGCTACTCCGTCCTA -3'	5'-GGTCATCTATGGGGCTTGAATCT -3'	
